# The selective reversible FAAH inhibitor, SSR411298, restores the development of maladaptive behaviors to acute and chronic stress in rodents

**DOI:** 10.1038/s41598-018-20895-z

**Published:** 2018-02-05

**Authors:** Guy Griebel, Jeanne Stemmelin, Mati Lopez-Grancha, Valérie Fauchey, Franck Slowinski, Philippe Pichat, Gihad Dargazanli, Ahmed Abouabdellah, Caroline Cohen, Olivier E. Bergis

**Affiliations:** 1 Sanofi, Strategy & Business Development, Chilly-Mazarin, France; 2 Sanofi R&D, Global Project Management, Immuno-Inflammation, Chilly-Mazarin, France; 3 Sanofi R&D, Neuroscience Therapeutic Area, Chilly-Mazarin, France; 4 Sanofi R&D, Integrated Drug Discovery, Chilly-Mazarin, France; 5 Sanofi R&D, Integrated Planning and Operations Management, Chilly-Mazarin, France; 6 Sanofi R&D, Global Project Team Neuroscience & Ophthalmology, Chilly-Mazarin, France; 7 Sanofi R&D, Translational In Vivo Models, Chilly-Mazarin, France

## Abstract

Enhancing endogenous cannabinoid (eCB) signaling has been considered as a potential strategy for the treatment of stress-related conditions. Fatty acid amide hydrolase (FAAH) represents the primary degradation enzyme of the eCB anandamide (AEA), oleoylethanolamide (OEA) and palmitoylethanolamide (PEA). This study describes a potent reversible FAAH inhibitor, SSR411298. The drug acts as a selective inhibitor of FAAH, which potently increases hippocampal levels of AEA, OEA and PEA in mice. Despite elevating eCB levels, SSR411298 did not mimic the interoceptive state or produce the behavioral side-effects (memory deficit and motor impairment) evoked by direct-acting cannabinoids. When SSR411298 was tested in models of anxiety, it only exerted clear anxiolytic-like effects under highly aversive conditions following exposure to a traumatic event, such as in the mouse defense test battery and social defeat procedure. Results from experiments in models of depression showed that SSR411298 produced robust antidepressant-like activity in the rat forced-swimming test and in the mouse chronic mild stress model, restoring notably the development of inadequate coping responses to chronic stress. This preclinical profile positions SSR411298 as a promising drug candidate to treat diseases such as post-traumatic stress disorder, which involves the development of maladaptive behaviors.

## Introduction

The endocannabinoid (eCB) system is formed by two G protein-coupled receptors, CB1 and CB2, and their main transmitters, N-arachidonoylethanolamine (anandamide; AEA) and 2-arachidonoyglycerol (2-AG)^1^. ECBs have an important neuromodulatory function in the periphery and in the central nervous system, regulating several physiological processes, such as appetite, cognition, anxiety, mood and pain^2–5^. Alterations in eCB signalling have been demonstrated in a wide range of pathological conditions including inflammation, immunological disorders, neurological and psychiatric conditions, obesity and metabolic syndromes and cancer (for recent reviews, see^6,7^). These findings have triggered significant interest in the development of eCB-interacting drugs, including direct-acting receptor ligands and catabolism inhibitors to treat these conditions^8^.

The signaling function of AEA is terminated by enzyme hydrolysis principally involving the serine hydrolase fatty acid amide hydrolase (FAAH)^9^. Highly selective, mostly irreversible inhibitors for FAAH with structural diversity and different interaction mechanisms within the FAAH active site have been described (for review see^10^). The most investigated FAAH inhibitor is URB597^11^, which was reported to display anxiolytic- and antidepressant-like activity in various rodent models^12,13^. Based on these findings, several irreversible FAAH inhibitors (e.g. BIA 10-2474, PF-04457845, JNJ-42165279) have entered into clinical trials to assess their potential efficacy in patients suffering from major depressive disorder (MDD), social anxiety or post-traumatic stress disorder (PTSD). However, several of these studies were either put on hold because of safety issues (e.g. BIA 10-2474) or terminated for strategic reasons (e.g. PF-04457845)^14^, so that no definitive conclusion could be drawn on the therapeutic potential of FAAH inhibitors against stress-related disorders. In the case of BIA 10-2474, the phase 1 trial led to the death of one volunteer and produced mild-to-severe neurological symptoms in four others. Although the cause of the clinical neurotoxicity is unknown, it has been postulated that off-target activities of BIA 10-2474 due to its irreversible nature may have played a role^15^, suggesting that reversible FAAH inhibitors may be safer.

**Figure 1:**
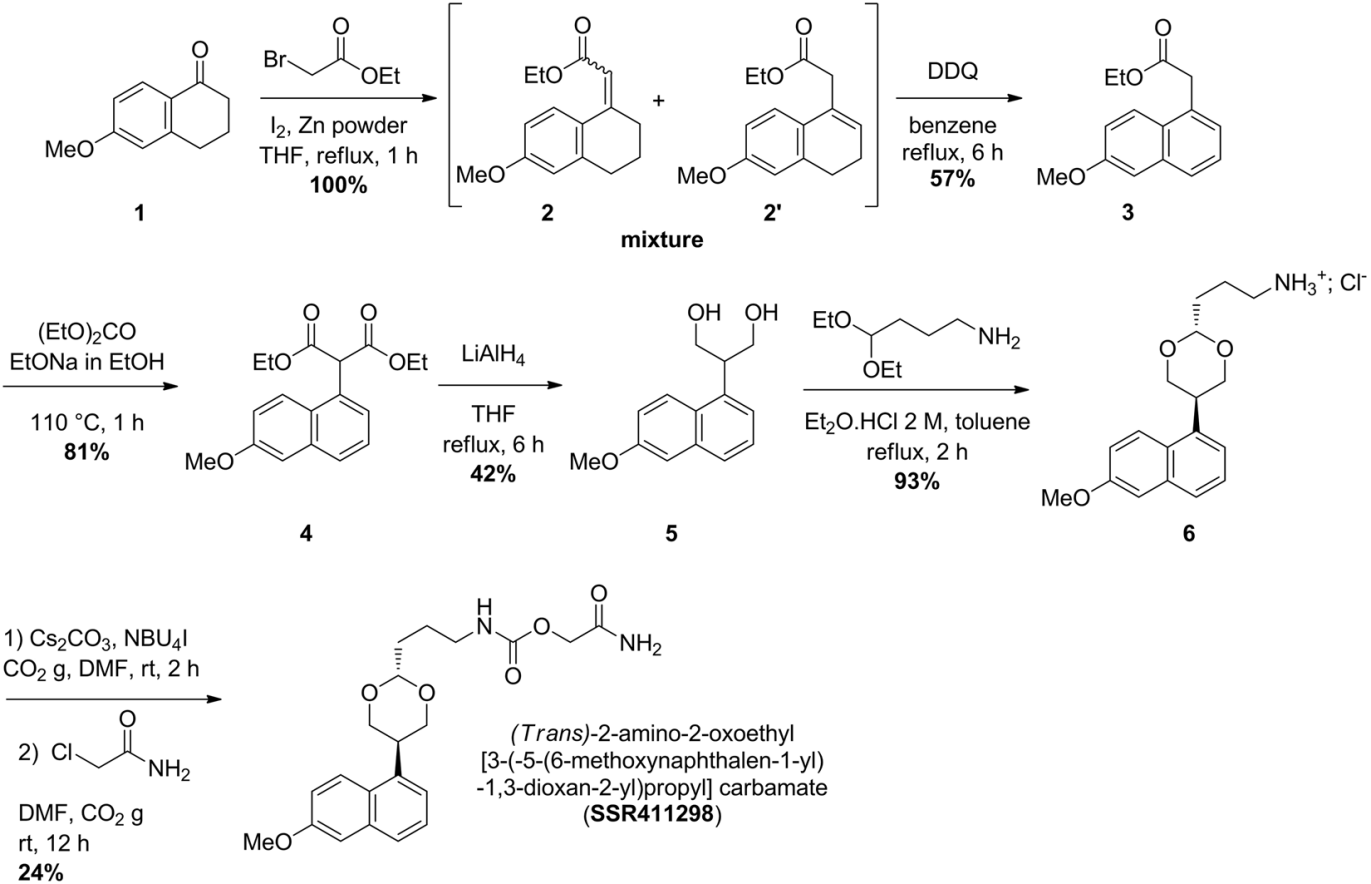
Synthesis of SSR411298. The compound was prepared in 24% yield by carbamation of (*trans*)-3-[-5-(6-methoxynaphtalen-1-yl)-1,3-dioxan-2-yl]propanamine (**6**) with chloroacetamide. Compound (**6**) was obtained *via* the dioxane formation of 2-(6-methoxynaphtalen-1-yl)propan-1,3-diol (**5**) with 4,4-diethoxybutanamine, in 93% yield. Compound (**5**) was isolated in 42% yield from the reduction of diethyl-2-(6-methoxynaphtalen-1-yl)propanedioate (**4**). Compound (**4**) was synthesized by alkylation of ethyl-(6-methoxynaphtalen-1-yl)-acetate (**3**) with ethylcarbonate in 81% yield. Compound (**3**) was prepared in two steps, in 57% overall yield, consisting in a substitution-deshydratation sequence of 6-methoxy-1,2,3,4-tetrahydronaphtalen-1-one (**1**) with ethyl bromoacetate, followed by an oxydation of the naphtalene ring by DDQ.

In the present study, we describe the characterization of a structurally distinct, potent, selective and reversible FAAH inhibitor, SSR411298 (2-amino-2-oxoethyl{3-[trans-5-(6-methoxy-1-naphthyl)-1,3-dioxan-2-yl]propyl} carbamate) (Fig. 1). More specifically, the effects of SSR411298 were evaluated in various animal models addressing different aspects of anxiety and depressive disorders. A second objective was to evaluate possible behavioral side-effects of SSR411298, more specifically related to the modulation of the eCB system, i.e. impairment in motor activity and coordination, catalepsy, nociception, physical dependence, and deficits in learning and memory.

## Methods and Materials

### Ethics statement

All experimental procedures described herein were carried out in accordance with the “Guide and Care and Use of Laboratory Animals” (National Institutes of Health) and were approved by the Animal Ethics Committee of Sanofi.

### Animals

Animals had access to food and water *ad libitum* with a 12-h light/dark cycle (lights on at 7:00 a.m.). The following species and strains were used: (1) mice: BALB/c, CD1, NMRI, OF1 and Swiss (Charles River Laboratories, Janvier Labs, Le Genest Saint Isle, France or Iffa Credo, Les Oncins, France); (2) Rats: Long Evans, Sprague-Dawley (Charles River Laboratories or Iffa Credo) and Wistar (Janvier Labs or Iffa Credo); (3) Gerbils: Mongolian (Janvier Labs) (see below for further details). Different species and strains were used on the basis of pilot experiments, which demonstrated that some species and/or strains are more suitable than others in certain models. Tests were performed during the light (day) cycle. During operant conditioning, rats were maintained at 85% of free-feeding body weight by restricting the daily ration of diet.

### Drugs

**Figure 2:**
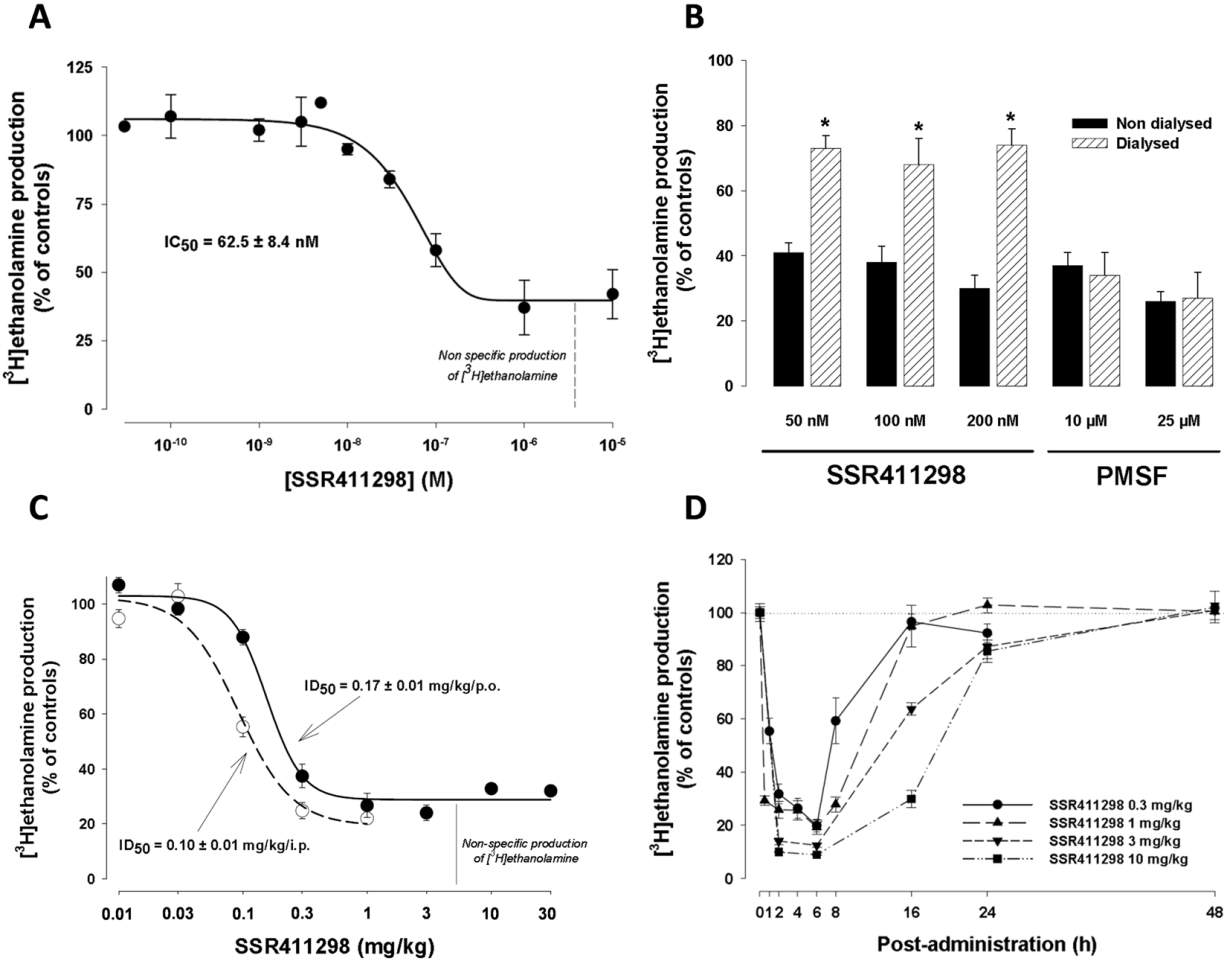
(**A**) Inhibitory effect of SSR411298 on mouse brain FAAH. Brain homogenates were incubated with 10 μM of [^3^H]anandamide in the absence (controls) or presence of increasing concentrations of SSR411298 (3 × 10^−11^ M to 10^−5^ M). Data are the mean ± SEM of 4 independent experiments; (**B**) Reversibility of the effects of SSR411298 and PMSF on mouse brain FAAH activity by dialysis. For each concentration, data are the mean ± SEM of mouse brain FAAH activity estimated by measuring [^3^H]ethanolamine production, expressed in percent of mean of controls after 16 to 18 hours at 25 °C without (black bars) or with dialysis (hatched bars). *P < 0.05 versus non dialyzed (Student’s t-test); (**C**) *Ex vivo* effect of SSR411298 on mouse brain FAAH activity 2 hours after intraperitoneal or oral administration; (**D**) Time-course of p.o. treatment with SSR411298 on mouse brain FAAH activity. Each point represents the mean ± SEM of 2 to 5 independent experiments. [^3^H]ethanolamine production are expressed as percent of mean of controls (vehicle treated mice). ID_50_ are calculated on specific [^3^H]ethanolamine production (equal to the total minus non-specific production).

SSR411298, diazepam, fluoxetine, rimonabant and WIN 55,212–2 mesylate (Sanofi Medicinal Chemistry), anandamide (Tocris Bioscience, Bristol, UK), Δ^9^-tetrahydrocannabinol (Δ^9^-THC), phenyl benzoquinone (PBQ) and phenylmethylsulfonyl fluoride (PMSF) (Sigma-Aldrich, Lyon, France or Saint-Louis, USA) were dissolved or suspended in distilled water with 0.6% methylcellulose and the addition of 5% Tween 80 (Sigma-Aldrich) or 2% cremophor in *in vivo* studies and suspended in DMSO at 10 mM in *in vitro* experiments. In the drug discrimination study, SSR411298 was prepared in 0.6% methylcellulose, 0.5% SDS and 0.01% dimethicone, and Δ^9^-THC was prepared in 7.12% ethanol, 7.12% cremophor and 87.76% of 0.9% NaCl. Doses refer to the weight of the free base. WIN 55,212–2 mesylate was dissolved in a few drops of polysorbate 80 and then diluted in saline (0.9% NaCl) solution; the final concentration of polysorbate 80 being 0.3%. SSR411298 was administered orally (*per os*, p.o.) in the behavioral tests with the exception of the anandamide-induce rotation test, the rimonabant challenge test to determine the occurrence of physical dependence, the chronic mild stress model and the separation-induced distress vocalization procedure where it was administered intraperitoneally (i.p.) or subcutaneously (s.c.). These administration routes were chosen because exploratory experiments showed that they are more suitable in these tests than the p.o. route. Moreover, in the experiments investigating the therapeutic potential of SSR411298 or its possible effects on learning and memory, the doses ranged from 0.3 to 30 mg/kg (p.o. or i.p.). This selection was based on the results from the *ex-vivo* experiment showing that SSR411298 produced maximal inhibition of FAAH between 0.3 and 30 mg/kg. We used higher doses (up to 500 mg/kg) in the drug discrimination and activity tests to determine a therapeutic window. Different treatment schedules were chosen because some of the procedures used required repeated administration to observe a drug effect (e.g. Morris water maze, forced-swimming, chronic mild stress, the rimonabant challenge test). Moreover, when SSR411298 was given acutely an injection time between 60 and 120 min was used because peak FAAH inhibition occurs between 60 and 360 min (see time-course in Fig. 2D). Volume of administration was 10 or 20 ml/kg in mice, 1 or 5 ml/kg in rats. All drug solutions were prepared fresh daily.

## Synthesis of SSR411298

The synthesis of SSR411298 was achieved as depicted in Fig. 1. 2-(6-methoxynaphthalen-1-yl)propan-1,3-diol (**5**) was prepared *via* a four steps sequence reported by Raizon *et al*.^16^ as follows: A solution of 6-methoxy-3,4-dihydronaphthalen-1(*2H*)-one (**1**, 50 g), ethyl bromoacetate (62 g), iodine (5 g) and zinc powder (33 g) in tetrahydrofuran (500 mL) was heated to 50 °C to initiate the reaction. The stirring was increased and the reaction mixture was refluxed for one hour under agitation. Then, the reaction mixture was cooled to room temperature and a saturated aqueous solution of potassium bisulfate was added until pH was adjusted between 1 and 2. The mixture was then refluxed one additional hour, filtered and the filtrate extracted with diethyl ether. The organic layers were gathered, dried over sodium sulfate, filtered and concentrated *in vacuo* to furnish 72 g (100% yield) of a mixture of crude ethyl 2-(6-methoxy-3,4-dihydronaphthalen-1(*2H*)-ylidene)acetate (**2**) and ethyl 2-(6-methoxy-3,4-dihydronaphthalen-1-yl)acetate (**2′**). 70 g of this mixture of **2** and **2′** was subjected to DDQ (2,3-dichloro-5,6-dicyano-1,4-benzoquinone) (74 g) in benzene (500 mL) for 6 hours under reflux. After 6 hours, the mixture was cooled down to room temperature, filtered and the filtrate was concentrated to dryness. The resulting residue was purified by flash chromatography to provide 39.4 g (57% yield) of ethyl 2-(6-methoxynaphthalen-1-yl)acetate (**3**). A solution of this latter (**3**, 14 g) in ethyl carbonate (46 mL) was heated to 110 °C, and a freshly prepared solution of sodium ethanolate in ethanol (1.45 g of sodium in ethanol) was added to this mixture in 45 minutes while distilling off the ethanol. The whole mixture was stirred one additional hour at 110 °C, then cooled down to room temperature, 100 mL of water were added, followed by an aqueous solution of HCl until pH = 1, extracted with dichloromethane (2 × 150 mL). The organic layers were gathered, dried over sodium sulfate, filtered and concentrated *in vacuo*. The resulting residue was purified by flash chromatography to provide 14.5 g (81% yield) of diethyl 2-(6-methoxynaphthalen-1-yl)malonate (**4**). To a solution of this malonate (**4**, 31 g) in tetrahydrofuran (150 mL) was added a solution of lithium aluminum hydride (LiAlH_4_, 7.5 g) in tetrahydrofuran (150 mL). The resulting mixture was refluxed for 6 hours, cooled down to room temperature and hydrolyzed by adding successively water (7.5 g), a 10% solution of sodium hydroxide (21 g) and once again water (7.5 g). The whole mixture was then stirred one hour at 50 °C, filtered, while washing the solid with hot tetrahydrofuran. The filtrate was concentrated to dryness, diluted with chloroform, and this solution was washed twice with water, dried over sodium sulfate, filtered and concentrated *in vacuo*. The resulting residue was purified by flash chromatography to give 9.5 g (42% yield) of 2-(6-methoxynaphthalen-1-yl)propane-1,3-diol (**5**).

(*trans*)-3-(5-(6-methoxynaphthalen-1-yl)-1,3-dioxan-2-yl)propan-1-amine hydrochloride (**6**) was prepared in one step reported by Dargazanli *et al*.^17,18^ as follows: To a solution of 2-(6-methoxynaphthalen-1-yl)propane-1,3-diol (**5**, 7.56 g) in toluene (300 mL) were successively added 4,4-diethoxybutan-1-amine (6.8 g) and 70 mL of a 2 M solution of HCl in diethyl ether. The whole mixture was then refluxed for 2 hours, cooled down to room temperature, and the precipitate was collected by filtration and washed with diethyl ether to provide 10.2 g (93% yield) of (*trans*)-3-(5-(6-methoxynaphthalen-1-yl)-1,3-dioxan-2-yl)propan-1-amine hydrochloride (**6**) as a brown solid. This latter was then used under its free base form for the preparation of SSR411298 reported by Abouabdellah *et al*.^19^ as follows: In a three-necks round-bottomed-flask, carbon dioxide (gaz) was bubbled for two hours in a solution of (*trans*)-3-(5-(6-methoxynaphthalen-1-yl)-1,3-dioxan-2-yl)propan-1-amine (free base of **6**, 20 g), cesium carbonate (64 g) and tetra-*n*-butylammonium iodide (73.14 g) in dimethylformamide (400 mL) under vigorous stirring at room temperature. Then, a solution of chloroacetamide (18.5 g) in dimethylformamide (70 mL) was added dropwise to the whole mixture while the carbon dioxide bubbling is maintained for five hours. The reaction mixture was stirred overnight, then filtered, and the resulting filtrate was concentrated under reduced pressure. The resulting residue was transferred in a separating funnel, diluted with water (250 mL) and ethylacetate (250 mL). The organic layer was then successively washed with an aqueous solution of 1 N HCl (150 mL), an aqueous saturated solution of sodium hydrogenocarbonate (150 mL), brine (150 mL), dried over sodium sulfate, filtered and concentrated *in vacuo*. The resulting residue was purified by flash chromatography with ethylacetate/methanol 95/5 to furnish the title compound which was finally crystallized in ethylacetate, giving 6.5 g (24 % yield) of pure (*trans*)-2-amino-2-oxoethyl-[3-(5-(6-methoxynaphthalen-1-yl)-1,3-dioxan-2-yl)propyl] carbamate (**SSR411298**). ^1^H nuclear magnetic resonance (400 MHz in DMSO-*d*_6_): (*trans* relative stereochemistry) 8.13 (d, *J* = 9.4 Hz, 1 H), 7.72 (d, *J* = 8.2 Hz, 1 H), 7.41 (t, *J* = 7.4 Hz, 1 H), 7.34 (d, *J* = 2.7 Hz, 1 H), 7.24 (br s, 1 H), 7.23 (s, 1 H), 7.22 (d, *J* = 4.0 Hz, 1 H), 7.20 (d, *J* = 2.7 Hz, 1 H), 7.13 (br s, 1 H), 4.73 (t, *J* = 4.7 Hz, 1 H), 4.31 (s, 2 H), 4.15 (dd, *J* = 10.8, 3.9 Hz, 2 H), 3.95 (t, *J* = 10.3 Hz, 2 H), 3.87 (s, 3 H), 3.86 (m, 1 H), 3.03 (dd, *J* = 12.8, 6.6 Hz, 2 H), 1.59 (m, 4 H); LC-MS ESI+ m/z 425.1692 [C_21_H_26_N_2_O_6_Na (M+Na) requires 425.1689]; mp 148–150 °C.

## Characterization of the mechanism of action of SSR411298

### Selectivity profile

The effect of SSR411298 on approximately 100 different human receptors, ion channels, enzymes and transporters (see Supplementary Material Table S1) was evaluated at a contract research organization (CEREP, Celle L’Evescault, France) using established protocols or through internal studies. IC_50_ were determined in cases where significant activity was observed at 10 μM (≥50% inhibition).

### Inhibition of FAAH activity in the mouse brain

#### Concentration response curve and reversibility of the effect *in vitro*

Male albino mice (OF1 strain) weighing 18–20 g were used. The method of Omeir *et al*.^20^ was used. FAAH activity was evaluated by measuring [^3^H]ethanolamine production after incubation of brain homogenates with anandamide tritiated on the ethanolamine moiety. Ten μl of brain homogenate (200 μg tissue per well) were incubated 20 minutes at 25 °C with substrate solution. The final volume of incubation, in polypropylene 96-well plates, was 70 μl in the incubation buffer. The reaction was stopped by addition of 140 μl of chloroform:methanol (2:1, v-v). After mixing and centrifugation, 30 μl of the aqueous upper phase containing the reaction product ([^3^H]ethanolamine) were mixed with 200 μl of scintillating cocktail (OptiPhase Supermix, PerkinElmer). The radioactivity was quantified (Wallac/PerkinElmer counter) and expressed as count per minute (CPM). First, the inhibitory effect of SSR411298 was studied by incubating mouse brain homogenate and 10 μM of [^3^H]anandamide in the absence (control) or presence of increasing concentrations of SSR411298 ranging from 3 × 10^−11^ M to 10^−5^ M. Four independent experiments were carried out. Second, the effects of SSR411298 on FAAH's kinetic constants Km and Vmax were studied. For this purpose, a constant pulse of [^3^H]anandamide (40–50 nCi) was added to increasing amounts of cold anandamide (0.1 to 200 μM). Samples were incubated for 20 minutes in the absence (control) or presence of SSR411298 (10, 20 or 50 nM). Nonspecific activity was obtained by incubating homogenates heated 15 minutes at 85–90 °C. Three independent experiments were carried out.

To investigate the inhibitory effect of SSR411298, control samples (mouse brain homogenates without SSR411298) were used. The radioactivity quantified in CPM for the control samples were averaged. Each sample was tested in duplicate and radioactivity values were transformed as percent of the mean of the control samples. IC_50_ values were determined from four independent dose-response curves on specific [^3^H]ethanolamine production, which was equal to the total production minus non-specific production. This latter corresponded to [^3^H]ethanolamine production which was not inhibited by SSR411298. The resulting IC_50's_ were then averaged (mean ± SEM). For each point of the kinetic study, specific [^3^H]ethanolamine production, expressed in CPM, was calculated by removing non-specific values to total production. Then they were corrected by (a) the volume of extraction (30 μl taken on 116.7 μl), (b) the efficiency of the chloroform/methanol extraction (0.574), and (c) the efficiency of the β-counter (0.317) and expressed in pmole/mg tissue/min. From each kinetic study, Km and Vmax values were determined using a non-linear fitting program. The respective Km/Vmax ratio, including those obtained in the absence of SSR411298, were then calculated and plotted as a function of the concentration of SSR411298. The four points obtained were fitted by linear regression, the intercept with abscissa-axis indicating the inhibitory constant Ki value. The Ki values from the 3 independent experiments were averaged (mean ± SEM). From the linear regression fitted on the Km/Vmax ratios of the 3 independent experiments, the slope was compared to 0 and the R2 was determined.

To investigate the reversibility of SSR411298, the method of Edgemond *et al*.^21^ was used. Male albino mice (OF1 strain) weighing 16–18 g were used. Diluted homogenate (200 μg of original frozen tissue per 10 μl) was incubated in the absence (control) or presence of SSR411298 at 50, 100 or 200 nM, or the non-selective FAAH inhibitor, phenylmethylsulfonyl fluoride (PMSF) at 10 or 25 μM for 30 minutes at 25 °C. FAAH activity was measured extemporaneously on the preparations. Four independent experiments were carried out. For each experiment, 2 or 3 control samples (mouse brain homogenates without compound) were tested. The radioactivity quantified in CPM for the control samples were averaged. Each sample was tested in triplicate and radioactivity values were calculated as percent of the mean of the control samples, which were averaged. For each concentration of the compound, transformed data of non dialyzed and dialyzed samples were compared and analyzed by a Student's t-test.

#### Dose-response and time-course of the effect *ex vivo*

To measure FAAH activity, the same method as described in the previous paragraph was applied. Male albino mice (CD1 strain) weighing 18–20 g were used. For ID_50_ (drug dose required to inhibit 50% of degradation of the radioligand) determination, mice (4 to 6 animals per group) were treated 2 hours prior to sacrifice by increasing doses of SSR411298: 0.01 to 1 mg/kg and 0.01 to 30 mg/kg for i.p. and p.o. administrations, respectively. In the time-course experiment, SSR411298 (0.3, 1, 3 or 10 mg/kg) was administered p.o. 30 minutes to 72 hours before sacrifice. Groups of 2 to 6 mice were used. For each experiment, control animals (vehicle-treated) were used. [^3^H]ethanolamine productions measured by radioactivity quantified in CPM for control animals were averaged. Each sample was tested in duplicate and radioactivity values were calculated as percent of the mean of the control samples. For the dose-effect experiment, ID_50_ values were determined from independent dose-response curves using a four parametric logistic model. The resulting ID_50_ were then averaged (mean ± SEM) for i.p. and p.o. administrations. In the time-course experiment, data were submitted to one-way ANOVA followed by a Dunnett's t-test, with an accepted level of significance of P < 0.01.

### Effects on endocannabinoid levels in mice

Male albino mice (OF1 strain) weighing about 27 g were used. The levels of anandamide (AEA), oleoylethanolamide (OEA), palmitoylethanolamide (PEA) and 2-arachidonoyl glycerol (2-AG) were measured in the hippocampus of mice treated p.o. with either vehicle or SSR411298 at 0.3, 1, 3 or 10 mg/kg. These N-acyl amides were selected on the basis of pilot experiments showing that PEA, OEA and, to a lesser extent, AEA were the most sensitive to FAAH inhibition. Animals were sacrificed 2 hours after drug administration and brains were immediately removed and dissected on ice to extract the hippocampus (right and left). Each sample was homogenized in 1 mL of heptane/isopropanol 3/2 containing 10 nM of AEAd8 (Internal standard for AEA, PEA, OEA) and 1 μM of 2AGd8 (Internal standard for 2-AG), centrifuged at 12 000 g for 7 minutes at 4 °C, thereafter 200 μL of supernatant were transferred into conic glass screw neck vials (12 × 32 mm, from Waters) and evaporated. The dried residue was dissolved in 150 μL of acetonitrile and 150 μL of H_2_O and then 100 μL were analyzed by online SPE-LC-MS/MS. The online SPE (Solid Phase Extraction) coupled to the LC (Liquid Chromatography coupled with Mass Spectrometry detection) was performed with a “Symbiosis” apparatus from Spark Holland using a C18 HD, 7 μM cartridge (2 × 10 mm as internal diameter × length) and a Hypersil gold C18 (1.9 μm, 50 × 2.1 mm) column respectively for the on line extraction and separation. Elution was performed at 0.25 mL/min with a ACN/H_2_O + ACNH_4_ 2 mM gradient, both solvent containing 0.1% of HCOOH (formic acid). The analytes were identified and quantified by a triple quadrupole mass spectrometry detector (Quantum Ultra from Thermo Electron Corporation) equipped with a HESI probe and working in the SRM mode (Selective Reaction Monitoring). The concentrations were calculated according to the external calibration method by comparing the ratio (area of the analytes/area of related internal standard) of the samples to the external standard calibration curve. The SRM mode was set up in order to follow the fragmentations occurring when selected protonated molecular ions MH+ (at m/z 348, m/z 326 m/z 300 and m/z 379 for AEA, OEA, PEA and 2-AG respectively) were submitted to Collision Induced Dissociation (CID), to produce either a protonated ethanolamine (m/z 62) in the case of AEA, OEA and PEA, or a loss of glycerol at m/z 287 in the case of 2-AG. AEA concentrations were expressed as pmol/g of tissue and OEA, PEA and 2-AG were expressed as nmol/g of tissue. Data on AEA, PEA and OEA were analyzed using a Kruskal-Wallis test (because of the heterogeneity of variances) for factor “treatment” followed by two-sided Kruskal-Wallis multiple comparisons test with Bonferroni-Holm correction for factor “treatment” versus vehicle-treated group.

### Effects of rotations induced by intra-striatal infusion of anandamide (AEA) in mice

Microinjection of cannabinoid receptor agonists into the striatum has been shown to induce rotations through activation of the striatonigral pathway and/or inhibition of the striatopallidal pathway^22,23^. The objective of this experiment was to determine whether SSR411298 is able to augment turning behavior of exogenously-administered AEA. Female albino mice (CD1 strain) weighing 25–27 g and 7 to 9-week-old were used. This study was performed by a well-trained experimenter who infused unilaterally AEA (0.1 to 10 ng/1 µl) and vehicle under light restraint. An injection point was assessed using the eyes and back of the skull as landmarks. The descending point of the needle was slightly internal and caudal to the right orbitus. The duration of injection was 2–3 seconds. We used a 5 μl Hamilton microsyringe and a 10 mm calibrated needle (final length below the skin: 3.5 mm). Infusing one µL of AEA in 2–3 seconds did not produce any overt symptoms with the exception of turning behavior. Exploratory work showed that a length of 3.5 mm below the skin does not damage the tissue. After injection, mice were placed individually in Plexiglas cages (10 × 10 × 15 cm). The number of complete contralateral rotations was recorded visually and cumulated over 3 periods of 2 min (2–4, 5–7, 8–10 min) post-injection of AEA. Groups of 11 to 12 mice were used. SSR411298 at 0.1, 0.3, 1, 3 or 10 mg/kg and vehicle (control group) were injected i.p. 30 minutes before intrastriatal infusion of AEA at 0.3 ng. The number of contralateral rotations observed at each of the 3 times point were cumulated. Data were analyzed using an ANOVA followed by Dunnett's t-test.

### Discriminative stimulus properties in rats

The purpose of this experiment was to evaluate the potential ability of SSR411298 to substitute for the cannabinoid receptor agonist WIN 55,212–2. The subjective effects of drugs in humans are critical in the abuse of psychoactive substances such as cannabis and the discriminative stimulus effects of drugs in non-humans are related to, and often predictive of, subjective effects in humans. Preclinical models of subjective drug effects include two-choice drug discrimination procedures in which animals are trained to discriminate between a drug and vehicle. One important feature of drug discrimination procedures is that only compounds which evoke a similar subjective state than the training drug will produce consistent responding on the drug-associated lever/hole^24–26^. Male Sprague-Dawley rats (290 to 310 g, 8 to 10-week-old on arrival) were trained to discriminate a dose of 0.25 mg/kg s.c. WIN 55,212–2 from vehicle using a standard, two nose poking holes, to the final fixed ratio 10, food-rewarded operant procedure. Thus, rats obtained a sucrose pellet (45 mg, Bio-Serv, Flemington, USA) each time they poked 10 times into the appropriate hole in the operant test chamber. Responses into one hole were rewarded in sessions that followed WIN 55,212-2 injection, and responses into the other hole were rewarded during the session following vehicle injection (see Supplementary Material Figure S1 for further details on the procedure). Daily sessions were 15 min in duration. Once trained, the animals were randomly allocated to one group for substitution/generalization according to a Latin Square design. They were given substitution tests with a range of p.o. doses of SSR411298 (10, 30, 100 or 500 mg/kg) or a dose of 2 mg/kg i.p. Δ^9^-THC or their respective vehicle, each being administered on a single occasion, using a Latin Square design. Test sessions were interspaced by at least 2 training sessions. SSR411298 was administered 4 hours before the beginning of the test. This administration time was chosen because the plasma C_max_ was achieved between 4 and 8 hours at 100 mg/kg, and between 4 and 24 hours at 500 mg/kg. Δ^9^-THC was given 35 min before the session. Results were expressed as the number of rats choosing the drug-associated hole during the substitution tests. Substitution occurred when at least 80% of the total session responses were emitted in the WIN 55,212-2-appropriate hole, or partial when the total responses emitted in the WIN 55,212-2-appropriate hole was at least 20% and less than 80%. Absence of substitution was considered when the total responses emitted in the WIN 55,212-2-associated hole were less than 20%. The average response rates after drug administrations were expressed as responses/second.

### Mouse tetrad experiments

This battery of tests was designed to assess potential cannabimimetic activity of psychoactive drugs^27^. SSR411298 was given p.o. to 7 to 9-week-old male OF1 mice weighing 25–30 g. The following four indices were assessed: (1) motor activity, including ambulation, muscle tone and motor coordination, (2) nociception, (3) catalepsy, and (4) hypothermia. Ambulation was measured in mice individually placed for one hour in a Digiscan animal activity cage (Omnitech Electronics, Colombus, USA) immediately after the administration of SSR411298 at 3, 10, 30 and 100 mg/kg, p.o. Each cage consisted of a 42 × 42 × 30.5 cm clear Plexiglas box with a grid of 16 infrared beams 2.5 cm apart from front to back and from side to side. Cages were connected to a Digiscan Analyser that transmitted the activity data to a microcomputer. Ambulation consisted of the total number of beam interruptions on the horizontal sensors during the 60-minute test period. Muscle tone was measured in the traction test where mice were suspended by the forepaws on a wire strand attached between 2 stands 30 minutes after the administration of SSR411298 at 10, 30 and 100 mg/kg, p.o. Mice must pull-up on the wire in 5 seconds over 10 trials. Motor coordination was evaluated in the rotarod test 30 minutes after the administration of SSR411298 at 10, 30 and 100 mg/kg, p.o. Here, mice were placed on a counter-turning running cylinder (13.5 turns/minute) for 2 minutes. The number of falls was counted. Nociception was measured in the PBQ stretching test 20 minutes after the administration of SSR411298 at 1, 3, 10, 30 and 100 mg/kg, p.o. In this assay, mice were individually placed in Plexiglas cages (10 × 10 × 15 cm) and administered with PBQ (2 mg/kg i.p.). PBQ induced abdominal torsions and contractions, which were defined as stretches. The number of stretches was visually recorded between 5 and 15 min post PBQ injection. Catalepsy was assessed in the ring test 30 minutes after the administration of SSR411298 at 10, 30 and 100 mg/kg, p.o. The apparatus consisted of a 5 cm wire ring attached to a stand elevated 17 cm above the surface. Each mouse was placed on the ring for 5 minutes during which the absence of all voluntary movement (with the exception of respiratory movements) was recorded. Variations in rectal temperature were measured by inserting a thermocouple probe 2.0 cm into the rectum 30, 60 and 120 minutes after the administration of SSR411298 at 1, 3, 10, 30 and 100 mg/kg, p.o. Data were analyzed with a one- or two-way (hypothermia) ANOVA followed, when appropriate, by a Dunnett’s test.

### Evaluation of signs of physical dependence after challenge with the CB1 receptor antagonist rimonabant in mice

A well-validated method to demonstrate physical dependence to cannabinoids is to precipitate physical withdrawal by challenging chronically-treated animals with an appropriate antagonist, here a selective CB1 receptor antagonist^28,29^. Male CD1 mice weighing 18–20 g were administered SSR411298 (20 mg/kg, i.p.), Δ^9^-THC (10 mg/kg, s.c.), or vehicle for 4 days, twice a day, between 09:00 and 11:00 a.m. and between 04:00 and 06:00 p.m. On the test day (day 5), animals received an acute challenge with rimonabant (10 mg/kg, i.p.) 4 hours after the last injection (i.e the 9^th^) of SSR411298, Δ^9^-THC or vehicle. Immediately thereafter, each mouse was isolated in a vertical Plexiglas cylinder (35 cm high, 20 cm diameter) and observed 5 minutes later for a 30-minute period. Saw bedding covered the floor to absorb urines and feces. The following behaviors were recorded manually by an experimenter unaware of the drug conditions: Scratches, licks, groomings, paw tremors (rapid lateral movement of the forepaws), stretches, chews were recorded every minute. In addition, the occurrence of ptosis and hunched posture were recorded. Since the data were not distributed normally or lacked homogeneity of their variance, the global analysis was performed using a Kruskal-Wallis test, followed by a multiple comparison test.

### Evaluation of potential effects on learning and memory

The experimental procedures used address various aspects of learning and memory processes in rodents, including spatial working memory (Morris water maze^30^), working memory (Y-maze^31^) and visual episodic memory (novel object recognition task^32^). These hippocampal-dependent tasks were selected on the basis of previous studies showing that the modulation of the eCB system may alter these types of memory (for a review, see^33^).

#### The Morris water maze in rats

Male Wistar rats weighing 245–292 g at the start of the study were used. The Morris water maze apparatus consisted of a PVC pool (1.5 m diameter × 0.60 m high), filled with thermostated water (28 ± 2 °C) to a depth of 0.35 m, with the addition of milk to render the water opalescent. A Plexiglas escape platform (12 cm diameter) was placed into the pool, 1 cm below the water surface and 10 cm from the wall. The test room contained several permanent extramaze cues such as flag, etc. on walls. A video-tracking camera (placed 2 m above the center of the pool surface) monitored the trajectory of the rat and the video signal was transmitted to a computer in an adjacent room and analyzed using the VIDEOTRACK® system (View Point Ltd, Champagne au Mont d’Or, France). The platform was placed at one of four possible cardinal locations NW, SE, NE, and SW, and NW for learning session 1, 2, 3 and 4, respectively. Each learning session consisted of four trials, with a maximal duration of 120 s and an intertrial interval of 30 s. Latency times (in seconds) to find the hidden platform were recorded during each trial of each learning session. If the rat located the platform within the maximum time allowed (120 s), it was left on the platform for 30 s. If the rat did not locate the platform within the time limit, it was gently placed on it for a 30-s period. At the start of each trial of each day, the rat was gently placed at the periphery of the maze, opposite of the platform (i.e. for the first trial E, for the NW quadrant). For each subsequent learning session, a similar cardinal rule was applied (i.e. N, W, N, and E for the SE, NE and SW, and NW quadrant, respectively). All rats received administrations of SSR411298 at 1, 3 and 10 mg/kg, p.o. or vehicle, 60 min before the first trial of each day. A two-way ANOVA (“Treatment” and “Trial”) with repeated measures on factor “Trial” was performed followed by a Winer analysis of factor “Treatment” for evaluating “Treatment” effect. In addition to evaluate time-course acquisition across day, a Winer analysis of factor “Trial” was performed followed when appropriate by a Dunnett's test to compare trials 2, 3 and 4 to the first trial.

#### The Y-maze test in mice

Male NMRI mice weighing 16–18 g were used. The Y-maze consisted of 3 arms in gray PVC in the shape of a Y. Arms were 28 cm long, 6 cm wide with walls 15 cm high. Movement was tracked manually using homemade software by an experimenter located in an adjacent room *via* a camera mounted directly above the maze. The animal was placed in an arm facing the center (Arm A) for 5 min. A correct alternation occurred when the animal moved to the other 2 arms without retracing its steps (i.e. Arm A to B to C). Movements such as ABA were incorrect. Based on the movement over the entire session, the percentage of correct alternations was calculated [i.e. (Total number of alternations x 100)/(Total number of arm entries - 2)]. SSR411298 was administered at 3, 10 and 30 mg/kg, p.o. 60 min prior to testing. Statistics performed on total arm entries and percentage alternation consisted of using a one-way ANOVA, followed by a post-hoc Dunnett’s test for individual comparisons.

#### The novel object recognition task in mice

Male Swiss mice weighing 20–22 g were used. The test apparatus was based on that described by Ennaceur and Delacour^32^ in rats and adapted for use in mice^34^. The apparatus consisted of a uniformly lit (20 lux) PVC enclosure (52 L × 52 W × 40 H cm) with a video camera positioned 160 cm above the bench. The objects to be discriminated were a metal triangle (3.3 cm height, 5.5 cm wide) and a plastic piece of construction game (3 cm height and 3 cm wide). The observer was located in an adjacent room fitted with a video monitoring system. The experiment consisted of 3 sessions. During the first session, mice were allowed to become familiar with the experimental environment for 2 min (S1). Time spent active (animal moving around with or without sniffing and exploration) was measured. Twenty-four hours later, the animals were placed in the same enclosure containing two identical objects for the amount of time necessary to spend 20 s exploring these two objects to a limit of 5 min (exploration was defined as the animal having its head within 2 cm of the object while looking at, sniffing, or touching it) (S2). After a delay of 60 min, mice were placed back into the enclosure with a previously presented familiar object and a novel object for a period of 5 min (S3 or recall session). Times spent exploring the familiar and novel objects were recorded. Following a 60-min delay, normal mice spent more time exploring the novel object compared to the familiar one during S3. This reflects the ability of the animal to remember the familiar object. SSR411298 was administered at 3, 10 and 30 mg/kg, p.o. 60 minutes prior to S2. Data (time exploring each of the two objects, in seconds) were analyzed with a two-way ANOVA, with the treatment and the object as the between factors, followed by a Winer analysis for comparing the time spent exploring the familiar versus the novel object for each treatment. Twelve animals per group were used.

## Characterization of SSR411298 in models predictive of therapeutic activity against anxiety and depressive disorders

### Effects in models of anxiety

The tests used were shown in previous studies to address different facets of anxiety responses and they are capable of responding to and differentiating anxiolytic drugs of different classes through specific profiles of effect on different measures. For example, the behaviors displayed by rodents in the punished drinking, light/dark, elevated plus-maze and social interaction tests are particularly sensitive to benzodiazepines, i.e., drugs used against generalized anxiety disorder (for an in-depth discussion, see^35^). Moreover, terminal defensive behaviors in the mouse defense test battery (i.e. defensive biting), avoidance responses of the open arms of the evelated plus-maze following social defeat, distress vocalizations in rat pups separated from their mother and memory impairment after stressful rat exposure are claimed to model certain aspects of acute and post-traumatic stress conditions^35–39^.

#### The punished drinking test in rats

This test is among the most widely used animal model of anxiety^35^. It’s first description as a model of anxiety by Vogel and colleagues demonstrated that it is a reliable procedure to measure anxiety-related behaviors in rodents, which is insensitive to drugs not modulating anxiety-related behaviors^40^. Male Sprague-Dawley rats weighing 205–245 g were deprived of water for 48 h before testing and were placed in cages with a stainless steel grid floor (MED Associates Inc., St Albans, VT). Each cage contained a drinking tube connected to a 50-ml burette filled with tap water. Trials were started when the rat’s tongue came in contact with the drinking tube for the first time. An electric shock (0.5 mA, 600 ms) was delivered to the tongue every 20 licks. The number of shocks (punished responses) was recorded during a 5-min period. SSR411298 was administered at 1, 3 and 10 mg/kg, p.o., and diazepam was administered at 3 mg/kg, p.o. 60 minutes prior to testing. Data were analyzed with a one-way ANOVA followed, when appropriate, by a Dunnett’s test.

#### Light/dark test in mice

Male BALB/c mice weighing 18–20 g were used. Earlier experiments showed that this strain is particularly suitable for investigating the anxiolytic-like effects of drugs in this procedure^41^. The homemade test apparatus is based on that described by Misslin *et al*.^42^. It consisted of two polyvinylchloride boxes (20 × 20 × 14 cm) covered with Plexiglas. One of these boxes was darkened. A desk lamp placed 20 cm above the lit box and a neon tube fixed on the ceiling provided the room illumination so that the light intensity in the center of the illuminated box was 1000 lux. An opaque plastic tunnel (5 × 7 × 10 cm) separated the dark box from the illuminated one. At the beginning of the experiment, a mouse was placed in the illuminated box, facing the tunnel. Recording started when the animal entered the tunnel for the first time. The apparatus was equipped with infrared beams and sensors capable of recording the following parameter during a 4-min period: time spent by mice in the lit box. Data were analyzed by nonparametric χ^2^ test followed by Kruskal-Wallis multiple comparisons test. Experiments were performed 60 min (diazepam: 2.5 mg/kg) or 120 (SSR411298: 3, 10 and 30 mg/kg) min after p.o. administration of the compounds.

#### Social interaction test in gerbils

Male Mongolian gerbils weighing 50–60 g and 7-week-old were used. The procedure is based on that described by File *et al*.^43^. Testing lasted 2 days: a first habituation session on day 1 followed 24 h later by the test. For the habituation session, gerbils were placed individually in a plastic box (30 × 30 × 20 cm) under bright light (300 lux) for a 10-min free-exploration period. The following day, all gerbils were injected with vehicle or one dose of the test compounds. SSR411298 (1, 3 and 10 mg/kg) or diazepam (0.1, 0.3 and 1 mg/kg) were administered p.o. 120 or 60 min prior to testing, respectively. Thereafter, two gerbils from the same weight and the same treatment group but different cages were placed together in the experimental cage for a 4′30-min observation period. Interaction time was recorded manually and consisted in active behaviors such as grooming, chasing and playing. Five couples of gerbils were tested per treatment group. Data (total duration of social interactions) from control gerbils and gerbils treated with SSR411298 or diazepam were compared using a one-way ANOVA followed by a one-sided upper Dunnett’s test as an increase in social interaction durations was expected for treated groups.

#### The mouse defense test battery

Ten-week-old male OF1 mice weighing 25–32 g were used. Previous studies showed that this strain is particularly suitable for investigating the anxiolytic-like effects of drugs in this model^44^. The test was conducted in an oval runway as previously described^45^. *Pretest*: Sixty (diazepam: 0.3, 1 and 3 mg/kg) or 120 (SSR411298: 3, 10 and 30 mg/kg) minutes after p.o. administration of the drugs, the mouse was placed into the runway for a 3-min familiarization period, in which locomotor activity (number of line crossings) was recorded. *The rat avoidance test*: Immediately after the 3-min familiarization period, the experimenter introduced a hand-held dead male Long Evans rat (370–375 g, killed by CO_2_ inhalation just before the beginning of the experiment) 5 times at one end of the runway and brought up to the mouse at a speed of approximately 0.5 m/s. Approach was terminated when contact with the mouse was made or the mouse ran away from the approaching rat. Flight was measured by the number of avoidances of 5 trials. *Chase/flight test*: The rat was then brought up to the mouse at a speed of approximately 2 m/s. A constant distance of 2 meters separated the rat and the mouse when the rat was introduced in the runway. Risk assessment was given by a measure of the number of stops (pauses in movement). The rat was removed after the chase was completed. *Forced contact in the straight alley*: By closing 2 doors (60 cm distant from each other), the runway was then converted to a straight alley in which the mouse was confined. The experimenter brought the rat into contact with the mouse in the straight alley. Approaches were directed quickly (within 1 second) towards the rat’s head. For each such contact, defensive aggression was measured by the number of bites by the mouse to the rat. Data concerning locomotor activity (number of line crossings), flight (number of avoidances), risk assessment (number of stops) and defensive aggression (number of bites) were subjected to analysis. Normality and homogeneity of variances were checked with Shapiro-Wilk and Levene tests, respectively. The “number of line crossing” parameter was transformed using a square root transformation to obtain a normally distributed parameter and subsequently analyzed with a one-way ANOVA. The other parameters were assessed with non-parametric Kruskal-Wallis tests followed in case of significant effects by post-hoc one-sided lower Kruskal-Wallis multiple comparison tests versus respective control groups, as decreases in the different variables (number of avoidances, stops and bites) were expected for treated groups.

#### Social-defeat stress-induced anxiety in the elevated plus-maze test in mice

Male CD1 mice weighing 20–29 g were used. Social defeat was used as a stressor before exposure to the elevated plus-maze. This stressor has been shown to produce significant anxiogenic-like activity without any physical signs of distress. The procedure was a modification of the technique described in the rat by Miczek^46^. A naïve mouse was placed in the cage of a resident male aggressor, which was selected for its high level of aggression. Social agonistic offensive and defensive behaviors were interrupted by the experimenter, and the intruder removed from the area when it displayed a submissive posture after being attacked. Thereafter, the intruder was returned to the resident cage for 60 min and placed in a cylindrical wire mesh enclosure to avoid physical contact or injury. At the end of the interaction period, the intruder mouse was placed on the central platform of the elevated plus-maze. The maze consisted of two open and two enclosed arms opposite to each other and connected to an open central area. To slightly increase open arm exploration, a rim (1 cm in height) surrounded the perimeter of these arms. During a 5-min observation period, the number of entries into each type of arm and the time spent in each arm were recorded. Mice displaying 3 or less entries both in open and closed arms were discarded from the study. Time spent in the open arms was considered as the main index of anxiety^47^. It was expressed as the mean percentage of time spent in open arms to total time spent in both open and closed arms. Mice were treated twice, 24 hours and 30 min before the beginning of the stress procedure. Three independent experiments were conducted, each of them being constituted of 3 different groups of mice:

- Stressed mice, vehicle treated
- Stressed mice treated with one dose of SSR411298 (1, 3 or 10 mg/kg, p.o.)
- Non-stressed mice, vehicle treated, which were not submitted to the stress procedure.

The Bartlett test was applied and since the hypothesis of homogeneity of variances was rejected, non-parametric tests were used. The three experiments were analyzed separately. For each experiment, vehicle group was compared to the vehicle stressed group (Wilcoxon test) in order to validate the experiment. If the difference appeared significant, the treatment effect was tested using the comparison between the treated- and vehicle-stressed groups (Wilcoxon test).

#### Separation-induced ultrasonic distress vocalizations in rat pups

Female Sprague-Dawley rats were obtained with 10 male pups (3- to 4-day-old, on the day of arrival). The procedure used was adapted from the technique described by Gardner^48^. SSR411298 was administered s.c. twice at 1, 30 and 10 mg/kg, 24 hours and 30 minutes before testing. Fluoxetine (1, 3 and 10 mg/kg) and diazepam (0.3, 1 and 3 mg/kg) were administered s.c. 30 minutes before testing. For injection purpose, rat pups were briefly separated from their mother and littermates. For testing, the pup was placed in a soundproof cage (30 × 30 × 15 cm). The Ultravox system (Noldus, Wageningen, The Netherlands) was used to record ultrasonic vocalizations (in the 40-kHz range). A modified ultrasound detector (Mini-3 bat model) connected to a microphone (positioned next to the pup) was used to transform ultrasonic sound into audible sound. The signal was then filtered and sent to a computer where the Ultravox software recorded each bout of distress call that lasted more than 10 ms, during 3 minutes. The drug effects on the number of distress calls emitted during a 3-minute period were analyzed using the Kruskal-Wallis test. In case of significance, comparisons between treated groups and respective control were carried out using a one-sided lower Kruskal-Wallis multiple comparisons test with Bonferroni-Holm correction versus control group as a decrease in the mean number of distress calls is expected for treated groups.

#### Stress-induced impairment in episodic memory in the object recognition test in mice

Male Swiss mice weighing 16–18 g were used. The apparatus is the same as described above. During the first session, mice were allowed to become familiar with the experimental environment for 2 min (S1). Time spent active (animal moving around with or without sniffing and exploration) was measured. Twenty-four hours later, the animals were placed in the same enclosure containing two identical objects for the amount of time necessary to spend 15 s exploring these two objects to a limit of 5 min (S2). During this acquisition session, a pair of rats was placed under the grid floor of the experimental enclosure. Mice and rats were able to see and smell each other, but no direct physical contact was possible. After a delay of 60 min, mice were placed back into the enclosure with a previously presented familiar object and a novel object for a period of 5 min (S3 or recall session). Times spent exploring the familiar and novel objects were recorded. Following a 60-min delay interval, non-exposed mice spent more time exploring the novel object compared to the familiar one during S3. This reflects the ability of the animal to remember the familiar object. This was in contrast to rat-exposed mice, which failed to discriminate both objects, indicating an impairment of short-term visual episodic memory. SSR411298 was administered at 1, 3 and 10 mg/kg, p.o. 60 minutes prior to S2. The time spent exploring each of the two objects (expressed in seconds) during the recall session was analyzed with a two-way ANOVA with repeated measures on the factor “object”, followed, when appropriate by a Winer analysis for comparing the time spent in exploring the familiar versus the novel object for each treatment group. These statistical analyses were performed on the ranked data. Ten animals per group were used.

### Effects in models of depression

We have used two experimental procedures widely used in antidepressant drug discovery, i.e. the forced swimming test and the chronic mild stress model. While the former has limited predictive validity it is generally used for the primary screening of potential new antidepressants because of its high throughput. The chronic mid stress model is often used at later-stage profiling and has increased translatability potential. For an in-depth discussion on these models in antidepressant drug discovery, see^49^.

#### Chronic mild stress (CMS) test in mice

Male BALB/c mice weighing 17–25 g and 6-week-old at the beginning of the experiment were used. Earlier studies demonstrated that this strain is particularly suitable for investigating the antidepressant-like effects of drugs in this model^50^. The CMS protocol is based on that used previously^51^ and consists of the sequential application of a variety of mild stressors, including restraint, forced swimming in warm (35 °C) water, water and or food deprivation, pairing with another stressed animal, each for a period of between 2 and 24 hours. The CMS procedure lasted 42 days. The physical state was measured according to a physical state scale attributing 3 points to well-groomed and clean animals, 2 points to animals with disorganized coat and 1 point to animals showing loss of fur and dirty fur, once a week over the 42-day CMS period. At the end of the CMS procedure, mice were tested in the mouse defense test battery (MDTB) to assess the impact of CMS on anxiety levels and stress coping in a particularly threatening situation (i.e., forced contact with a Long Evans rat). The MDTB procedure was the same as described above. Here, flight (number of avoidances of the threatening rat), defensive aggression (i.e., biting by the mouse upon forced contact with the rat) and locomotor activity (before the rat exposure) were presented as the most relevant variables and were analyzed therefore. The administration of SSR411298 (10 mg/kg, i.p., once a day) or fluoxetine (10 mg/kg, i.p., once a day) started 15 days after the beginning of the stress exposure and lasted until the MDTB was completed (in total, 33 days of treatment).

Physical state data (expressed on a physical state scale from 1 to 3) from control stressed and unstressed animals were analyzed for each week of the CMS by a Wilcoxon test. Subsequently, physical state data from the 3 groups of stressed/treated animals were compared using a Kruskal-Wallis test followed by Kruskal-Wallis one-sided upper multiple comparisons tests with Bonferroni-Holm correction versus stressed control group (as increases in physical data scores are expected for mice treated with either fluoxetine or SSR411298). In the MDTB, the number of line crossings from control stressed and non-stressed animals was analyzed with a Student’s test. For treated stressed mice a one-way ANOVA was performed followed in case of significant effect by a one-sided lower Dunnett’s test versus stressed control group. Other data (avoidance frequency and bitings) from control stressed and non-stressed animals in the MDTB were analyzed with a Wilcoxon test. For each variable, a Kruskal-Wallis test was performed and followed by Kruskal-Wallis one-sided lower multiple comparisons tests with Bonferroni-Holm correction versus stressed control group, as decreases in the number of avoidances or bites were expected for mice treated with either fluoxetine of SSR411298.

#### Forced-swimming test in rats

Male Wistar rats weighing 235–290 g were used. The procedure has been described initially by Porsolt *et al*.^52^. Rats were placed in individual glass cylinders containing water (23 ± 1 °C). Two swimming sessions were conducted (an initial 15-minute pretest (training) followed 24 hours later by a 6-min test). The total duration of immobility (in seconds) was measured during the 6-min test period. The animal was judged to be immobile whenever it remained floating passively in the water. SSR411298 was administered at 0.3, 1 and 3 mg/kg, p.o., twice a day during 2 days, 60 minutes before pretest, immediately after, and 60 minutes before the second session of testing. Fluoxetine was administered at 3, 10 and 30 mg/kg, p.o., twice (immediately after the first session, and 60 minutes before session 2) in an independent study. Immobility time (in seconds) was assessed with one-way ANOVA. Subsequent comparisons between control group and treated groups were carried out using a one-sided lower Dunnett's test as a decrease in immobility time was expected for treated groups.

### Data availability

The datasets generated during and/or analysed during the current study are available from the corresponding author on reasonable request.

## Results

### Synthesis of SSR411298

SSR411298 was synthesized in one step from compound **6** as outlined in Fig. 1, using a protocol described by Abouabdellah *et al*.^19^. The preparation of intermediate **6** was realized in one step from **5** following procedures described by Dargazanli *et al*.^17,18^. Compound **5** was prepared in four steps from **1** as described by Raizon *et al*.^16^. The spectral data of SSR411298 were consistent with its structure.

### Characterization of the mechanism of action of SSR411298

#### Selectivity profile

When SSR411298 was tested at a concentration up to 10 μM for inhibition of radioligand binding to a battery of neurotransmitter (including CB1 and CB2) and peptide receptors, kinases, transporters and ion channels, results revealed a lack of interaction with any of these targets (data shown in Table S1).

### Inhibition of FAAH activity in the mouse brain

#### Concentration response curve and reversibility of the effect in vitro

SSR411298 inhibited mouse brain FAAH in a concentration-dependent manner; the maximal effect being reached at 1 μM (Fig. 2A). The IC_50_ value calculated from four independent experiments is 62.5 ± 8.4 nM. In the reversibility experiment, SSR411298 and PMSF inhibited mouse brain FAAH in a concentration-dependent manner after a 30-min incubation period: 29, 22, and 16% of controls for 50, 100 and 200 nM of SSR411298, and 28 and 19% of controls for 10 and 25 μM of PMSF, respectively (Table 1). After 16 to 18 hours of dialysis, the effects of SSR411298 significantly declined as compared to non dialyzed conditions (74 versus 30% for SSR411298 at 200 nM (Table 1 and Fig. 2B). In contrast, the effect of the irreversible FAAH inhibitor, PMSF, remained unchanged whether under dialyzed or non dialyzed conditions (27 versus 26% for PMSF 25 μM) (Table 1 and Fig. 2B).

**Table 1:** Study of the reversibility by dialysis of the effects of SSR411298 and PMSF on mouse brain FAAH activity.

Treatment	Concentration	[^3^H]ethanolamine production (% control)		
		T = 0	T = 16 to 18 hours	
			Non dialyzed	Dialyzed
Control		100 ± 2	100 ± 2	100 ± 3
SSR411298	50 nM	29 ± 4	41 ± 3	73 ± 4*
	100 nM	22 ± 3	38 ± 5	68 ± 8*
	200 nM	16 ± 3	30 ± 4	74 ± 5*
PMSF	10 µM	28 ± 7	37 ± 4	34 ± 7
	25 µM	19 ± 4	26 ± 3	27 ± 8

Mouse brain homogenates were incubated 30 minutes at 25 °C without (control) or with SSR411298 or PMSF. FAAH activity, estimated by [^3^H]ethanolamine production, was measured immediately after incubation (T = 0) and after 16 to 18 hours at 25 °C without (non dialyzed) or with dialysis (dialyzed). Data expressed as the percent of mean of control values, are the mean ± SEM of 3 to 4 independent experiments corresponding to a total of n dialysis cassettes. *P < 0.05 versus non dialyzed (Student’s t-test).

#### Dose-response and time-course of the effect ex vivo

Intraperitoneal or p.o. administration of SSR411298 produced a dose-dependent inhibition of brain FAAH activity (Fig. 2C) with ID_50s_ of 0.10 ± 0.01 and 0.17 ± 0.01 mg/kg, respectively. The time-course experiment showed that SSR411298 reduced significantly (P < 0.01) FAAH activity 2 hours post-administration, regardless the dose tested (Fig. 2D). These effects remained stable up to 6 hours before steadily declining until 24 hours post-administration, at which time point [^3^H]ethanolamine production in the treated groups was no longer statistically different from that of the control group.

### Effects on endocannabinoid levels in mice

**Figure 3:**
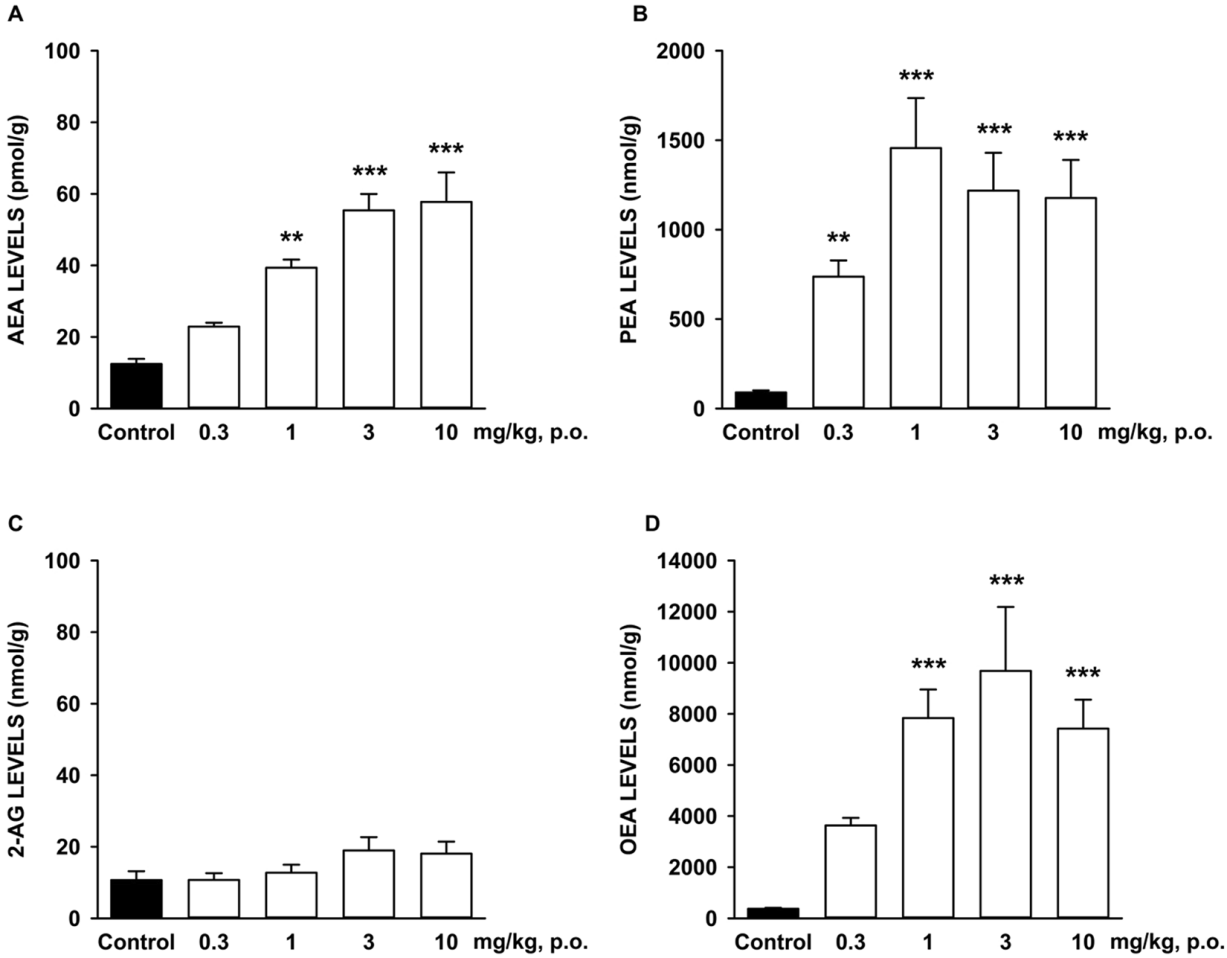
Effects of SSR411298 on levels of (**A**) anandamide (AEA), (**B**) palmitoylethanolamide (PEA), (**C**) 2-Arachidonoyl glycerol (2-AG) and (**D**) oleoylethanolamine (OEA) in the hippocampus of mice. Data represent mean ± SEM. *P < 0.05; **P < 0.01;***P < 0.001 (Kruskal-Wallis multiple comparison test versus vehicle treated group). N = 8 mice per group.

The levels of AEA, OEA, PEA and 2-AG were measured in the hippocampus of mice treated p.o. with SSR411298 at 0.3, 1, 3 or 10 mg/kg two hours prior to sacrifice. SSR411298 produced a significant global increase of tissular AEA, PEA and OEA levels in the hippocampus (KW analyzes: P < 0.0001 for all three ECs), but not of 2-AG levels (P = 0.1153). For AEA, PEA and OEA, the effects were dose-dependent and reached a plateau at 1 mg/kg for PEA and OEA, and at 3 mg/kg for AEA with peak levels of 1218.10 (PEA) and 9683.51 (OEA) nmol/g, and 55.40 pmol/g for AEA (Fig. 3A–D).

### Effects of rotations induced by intra-striatal infusion of anandamide (AEA) in mice

**Figure 4:**
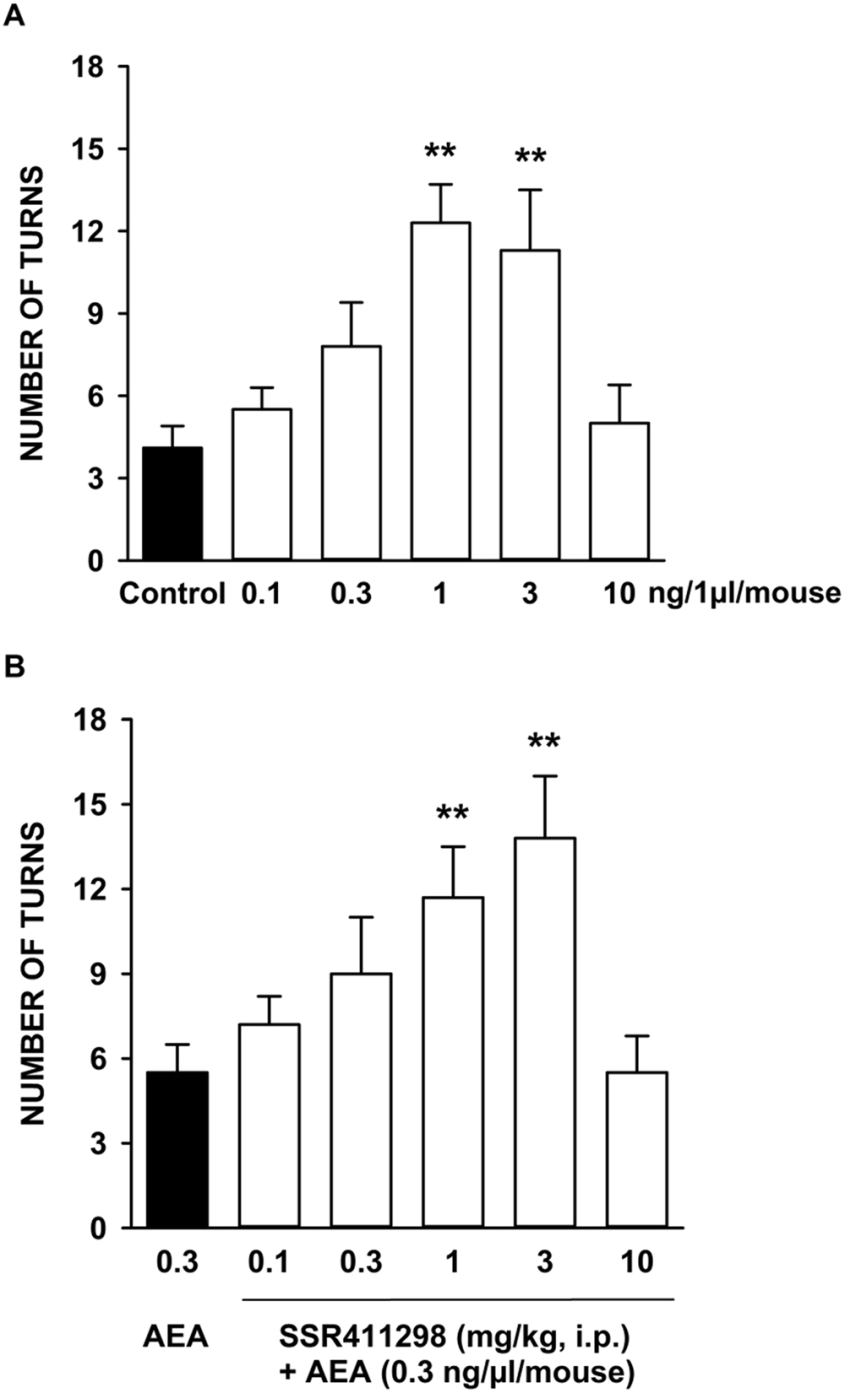
Potentiation by SSR411298 of turning behavior induced by intra-striatal infusion of anandamide (AEA) in mice. (**A**) Dose-response of AEA; (**B**) Potentiation by SSR411298 of the effects of AEA. Data represent the mean ± SEM. **P < 0.01 (Dunnett's t-test) vs. control or AEA-treated group. N = 12 to 24 mice per group.

Intrastriatal injection of anandamide (0.1 to 10 ng) produced a dose-dependent increase in contralateral turns [F(5,65) = 5.48, P < 0.001], an effect which reached statistical significance at 1 and 3 ng (Fig. 4A). At the highest dose (i.e. 10 ng), the effects on turning were no longer apparent. When given in association with a subactive dose of anandamide (i.e. 0.3 ng), SSR411298 potentiated significantly anandamide-induced turns at 1, 3 but not at 10 mg/kg [F(5,90) = 4.22, P < 0.002]) (Fig. 4B).

### Discriminative stimulus properties in rats

**Figure 5:**
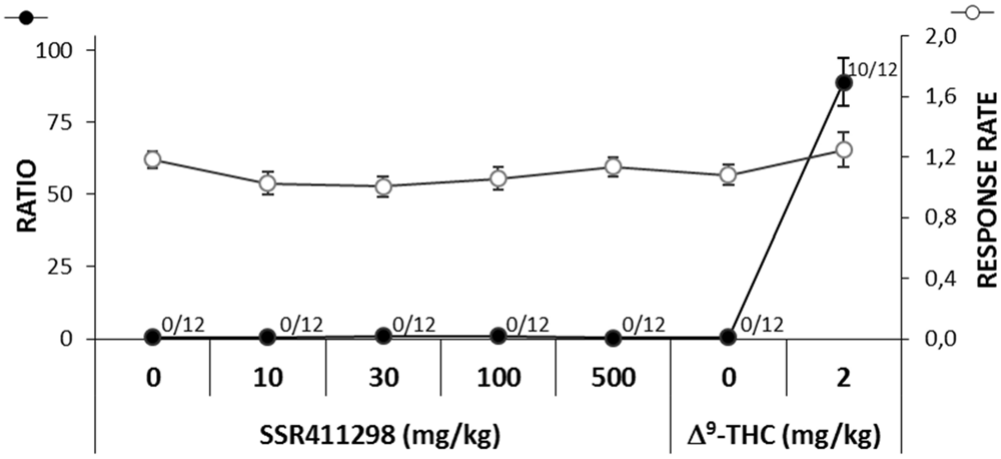
Effect of SSR411298 and Δ^9^-THC during generalization sessions in a drug discrimination procedure. The ratio represents the percentage of responses on the WIN 55,212-2-associated hole (●). Response rate is the number of responses per second (○). The number alongside each (●) symbol indicates the number of animals with complete generalization to WIN 55,212-2. SSR411298 at 10, 30, 100 and 500 mg/kg produced no generalization to WIN 55,212-2 whereas Δ^9^-THC produced complete generalization to WIN 55,212-2 in 10/12 rats. Data represent the mean ± SEM. N = 12.

The results are presented in Fig. 5. Δ^9^*-THC*: As expected, the vehicle of Δ^9^-THC produced no generalization/substitution to WIN 55,212–2. All the rats responded nearly exclusively on the vehicle of WIN 55,212-2-associated hole with 574.9 ± 7.5 responses versus 3.2 ± 0.9 on the other hole. The average percent of total responses emitted on the WIN 55,212-2-associated hole was 0.6 ± 0.2%. The mean response rate was 1.1 ± 0.1 response/s. In contrast, Δ^9^-THC at 2 mg/kg produced complete generalization/substitution to WIN 55,212-2 in 10/12 rats, partial generalization in 1/12 rat and no generalization in 1/12 rat. The mean of total responses emitted on the WIN 55,212-2-associated hole was 512.3 ± 50.9 versus 61.3 ± 45.8 on the other hole. The average percent of total responses emitted on the WIN 55,212-2-associated hole was 88.9 ± 8.3%. Δ^9^-THC at 2 mg/kg did not affect the mean response rate, 1.2 ± 0.1 response/s. *SSR411298*: The vehicle of SSR411298 produced no generalization/substitution to WIN 55,212-2. All the rats responded almost exclusively on the vehicle of WIN 55,212-2-associated hole with 575.2 ± 8.5 responses versus 2.8 ± 0.9 on the other hole. The average percent of total responses emitted on the WIN 55,212-2-associated hole was 0.5 ± 0.1%. The mean response rate was 1.2 ± 0.1 response/s. SSR411298 at 10, 30, 100, or 500 mg/kg produced no generalization/substitution to WIN 55,212-2. All the rats responded almost exclusively on the vehicle of WIN 55,212-2-associated hole. The average percent of total responses emitted on the WIN 55,212-2-associated hole were 0.6 ± 0.2, 0.9 ± 0.3, 1.0 ± 0.4 and 0.3 ± 0.1%, respectively. SSR411298 at 10, 30, 100 and 500 mg/kg did not affect the mean response rate, 1.0 ± 0.1, 1.0 ± 0.1, 1.1 ± 0.1 and 1.1 ± 0.1 response/s, respectively.

### Mouse tetrad experiments

**Figure 6:**
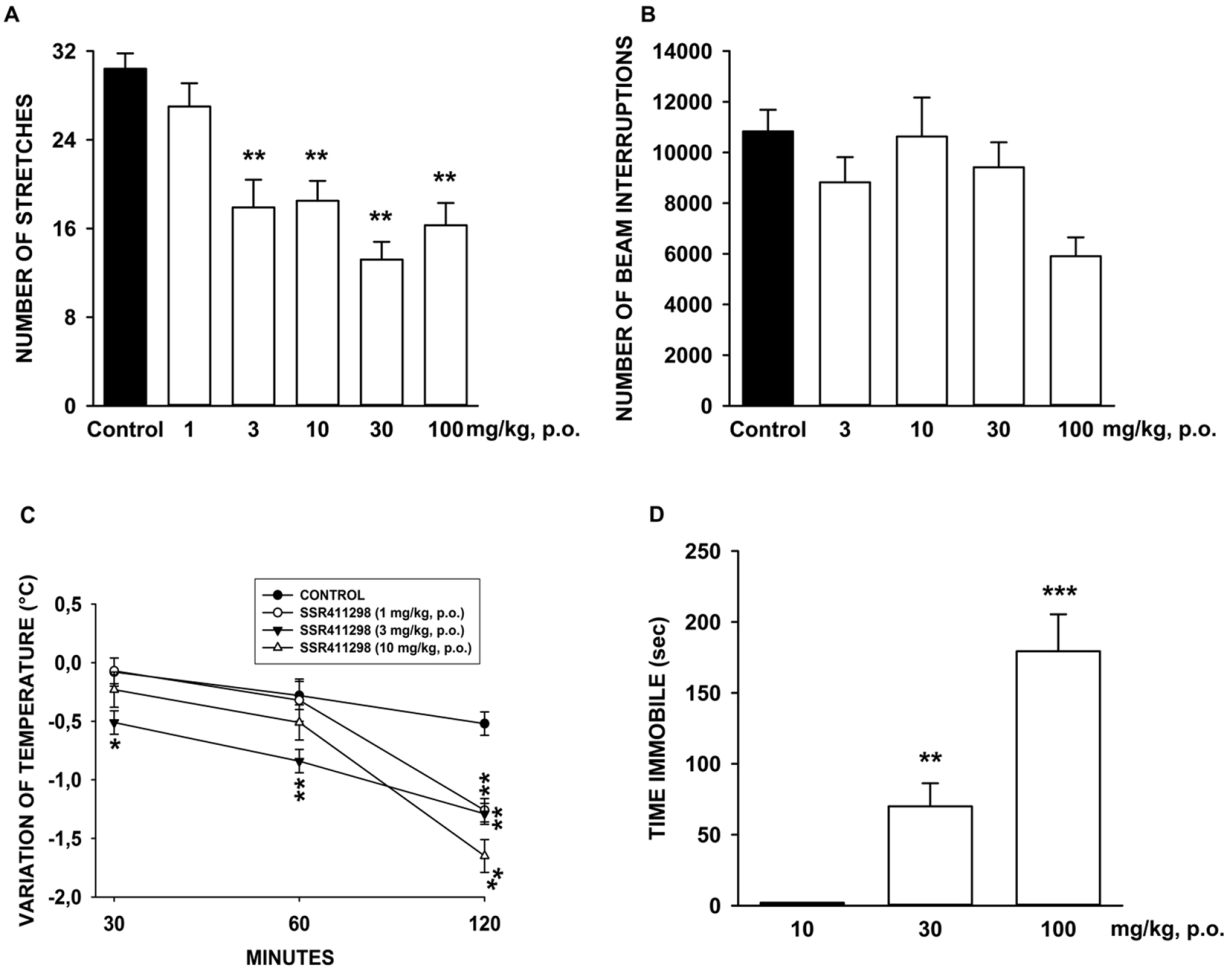
Study of the potential cannabimimetic activity of SSR411298. (**A**) PBQ: number of stretches; (**B**), Ambulation: number of beam interruptions on the horizontal sensors; (**C**) Temperature: °C; (**D**) Ring immobility: time immobile in seconds. **P < 0.01, ***P<0.001. Data represent the mean ± SEM. N = 8 to 20.

SSR411298 attenuated significantly stretching behavior elicited by PBQ from 3 mg/kg [F(5,74) = 13.86, P < 0.001] (Fig. 6A), decreased ambulation at 100 mg/kg [F(4,55) = 3.49, P < 0.01] (Fig. 6B) and produced hyperthermia at 1 mg/kg during the 120 minutes recording period, and at 3 and 10 mg/kg 120 minutes after its administration [2-way ANOVA: F(6,108) = 3.96, P < 0.001] (Fig. 6C). The drug produced catalepsy at 30 and 100 mg/kg [F(2,21) = 25.85, P < 0.001] (Fig. 6D). Finally, SSR411298 did not modify muscle tone and motor coordination up to 100 mg/kg as assessed in the traction and rotarod tests, respectively (data not shown).

### Evaluation of signs of physical dependence after challenge with the CB1 receptor antagonist rimonabant in mice

Global Kruskal-Wallis analyzes revealed a significant treatment effect for paw tremor [KW = 27.0, P < 0.001], stretch [KW0 = 26.9, P < 0.001], ptosis [KW = 21.2, P < 0.001], chew [KW = 14.1, P < 0.001], hunched posture [KW = 20.1, P < 0.001], scratch [KW = 26.6, P < 0.001], grooming [KW = 11.9, P <  0.01] and lick [KW = 13.2, P <  0.01]. Further analysis showed that the Δ^9^-THC group differed from the two other groups by a high occurrence of the following behaviors: paw tremor, stretch, ptosis, chew and hunched posture. Δ^9^-THC-treated mice exhibited fewer scratch and grooming behaviors as compared to vehicle-treated animals. SSR411298-treated animals did not differ from vehicle-treated mice on any of these behaviors (Table 2).

**Table 2:** Rimonabant-precipitated withdrawal signs in mice treated with SSR411298 or Δ^9^-THC.

	Control	SSR411298 (20 mg/kg, i.p.)	Δ^9^-THC (10 mg/kg, s.c.)
Paw tremor	7,79 ± 2,28	12,20 ± 2,62	91,31 ± 11,58**
Stretch	2,64 ± 1,99	1 ± 0,37	24,31 ± 4,31**
Ptosis	0 ± 0	0 ± 0	3,85 ± 1,53*
Chew	0,79 ± 0,30	1,20 ± 0,38	4 ± 0,90**
Hunched posture	0,57 ± 0,44	0,33 ± 0,16	7,15 ± 2,09**
Scratch	339,21 ± 49,13	288,27 ± 42,59	23,15 ± 4,77**
Lick	44,57 ± 4,58	55,47 ± 4,07	30,46 ± 3,91
Grooming	13,79 ± 1,62	13,27 ± 2,03	5,23 ± 1,75**

Treatment schedule: 2 injections/day/4 days. On day 5, the drugs were administered 4 hours after the last injection of rimonabant (10 mg/kg i.p.). Data represent mean ± SEM. *P < 0.05, **P < 0.01. N = 13 to 14.

### Evaluation of potential effects on learning and memory

#### The Morris water maze in rats

**Figure 7:**
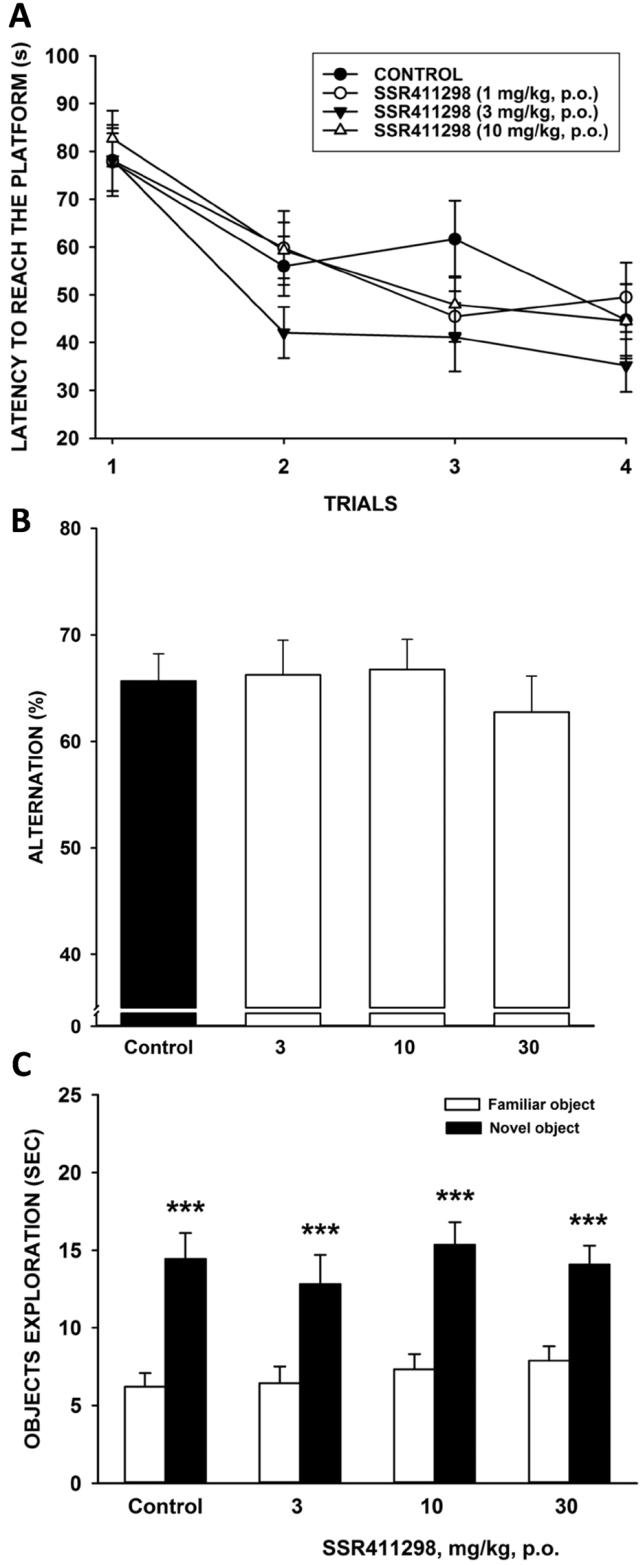
Effects of SSR411298 on (**A**), spatial reference memory in the Morris water maze in rats. Each line represents the average (±SEM) latency to reach the platform across trial. N = 10 rats per group; (**B**) episodic memory in the object recognition test in mice. Each bar represents the average (+SEM) time spent exploring a familiar or a novel object. The interval between the acquisition and the recall session was 60 min. ***P < 0.001 novel vs familiar object at the considered treatment condition. N = 12 mice per group; (**C**) spatial working memory in the Y-maze test in mice. N = 12 mice per group.

A two-way ANOVA on variable “Latency” revealed a significant “Trial” effect [F(3,108) = 31.696, P < 0.0001], no significant “Treatment” effect [F(3,36) = 1.169, P = 0.3349] and no significant “Treatment” × “Trial” interaction [F(9,108) = 0.964, P = 0.4740] (Fig. 7A shows the cumulated scores for each day). Winer analyses on variable “Latency” and factor “Treatment” for each level of factor “Trial” indicated that SSR411298-treated animals did not display altered performance throughout the experiment. Moreover, Winer analyses on variable “Latency” and factor “Trial” for each level of factor “Treatment” showed that acquisition performance in SSR411298-treated rats were not significantly different from vehicle-treated animals at any dose. Post-hoc Dunnett’s test confirmed that performance of rats in both vehicle- and SSR411298-treated groups improved across trials, indicating no alteration in working memory function.

#### The Y-maze test in mice

Statistical analysis did not show a global significant effect of treatment [F(3,44) = 0.35, P = 0.79] following the administration of increasing doses of SSR411298 (3, 10 and 30 mg/kg) indicating that the drug had no effect on memory performance in this test (Fig. 7B).

#### The novel object recognition task in mice

Using a procedure where the two objects were presented 60 minutes after the exposure to the familiar object, control mice spent more time exploring the novel one (14.5 ± 5.7 versus 6.2 ± 3.0 s) [ANOVA on factor “Objects”: F(1,44) = 94.9, P < 0.0001]. The preference for the novel object was not abolished by SSR411298 at 3, 10 or 30 mg/kg immediately after presentation of the familiar object [ANOVA on factor “Treatment”: F(3,44) = 0.49, P = 0.69, and “Treatment” x “Objects” interaction: F(3,44) = 0.53, P = 0.67] (Fig. 7C).

## Characterization of SSR411298 in models predictive of therapeutic activity against Anxiety and Depressive disorders

### Effects in models of anxiety

#### The punished drinking test in rats

**Figure 8:**
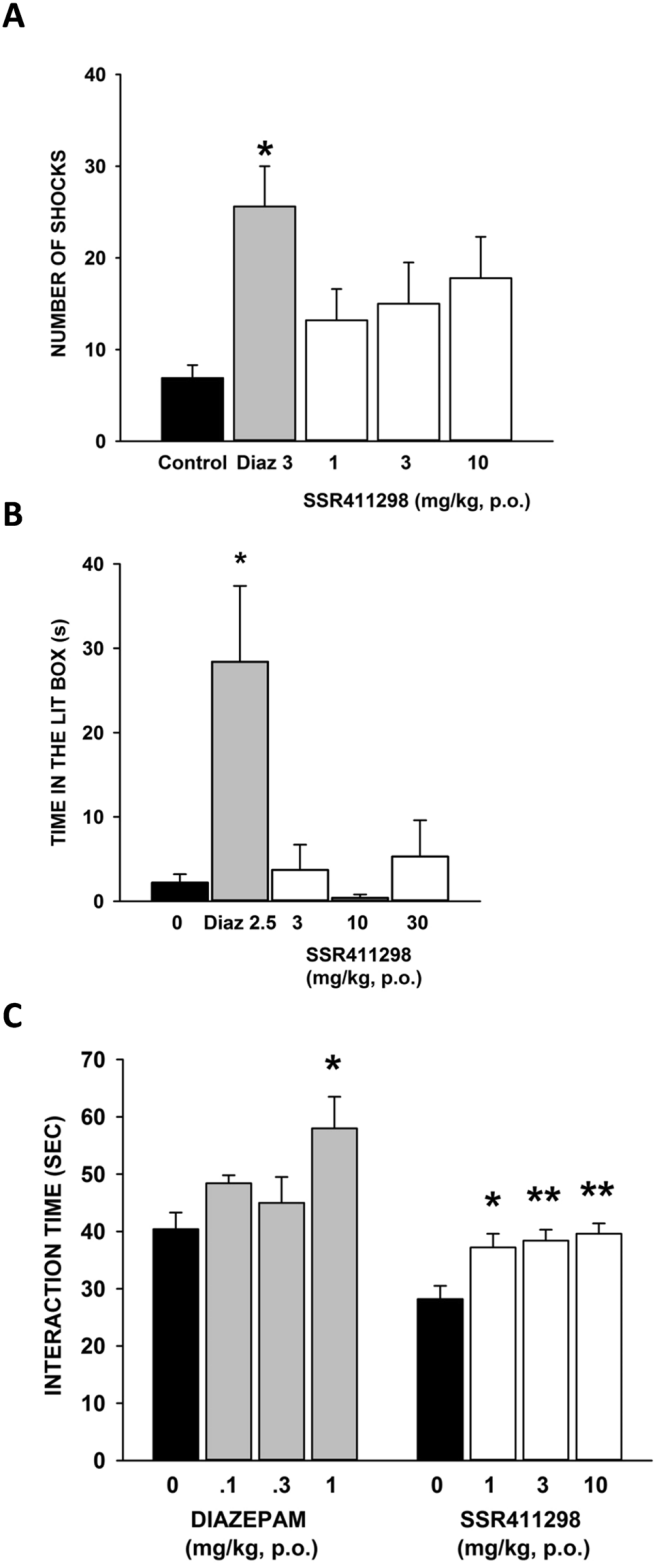
Effects of SSR411298 and diazepam in several classical models of anxiety, including the (**A**), punished drinking test in rats (N = 11–12 rats per group); (**B**) the light/dark test in mice (N = 11–12 mice per group) and (**C**), the social interaction in gerbils (N = 5 gerbils per group). Data represent mean + SEM, *P < 0.05, **P < 0.01.

ANOVA showed a global significant effect [F(4,54) = 2.71, P < 0.05]. Post-hoc test revealed that diazepam at 3 mg/kg, but not SSR411298 at any of the doses tested (i.e, 1, 3 and 10 mg/kg), significantly increased punished responding (Fig. 8A).

#### Light/dark test in mice

SSR411298 did not significantly modify the time spent in the bright cage over the dose-range tested (3 to 30 mg/kg). This was in contrast to the prototypical anxiolytic compound, diazepam, which produced a significant increase in this measure at 2.5 mg/kg [KW = 15.44, P < 0.01] (Fig. 8B).

#### Social interaction test in gerbils

Results showed that SSR411298 significantly increased the duration of social contacts [F(3,16) = 6.13, P = 0.0056] at 1, 3 and 10 mg/kg. The reference anxiolytic, diazepam, increased significantly this behavior at 1 mg/kg [F(3,16) = 3.65, P = 0.035] (Fig. 8C).

#### The mouse defense test battery

**Figure 9:**
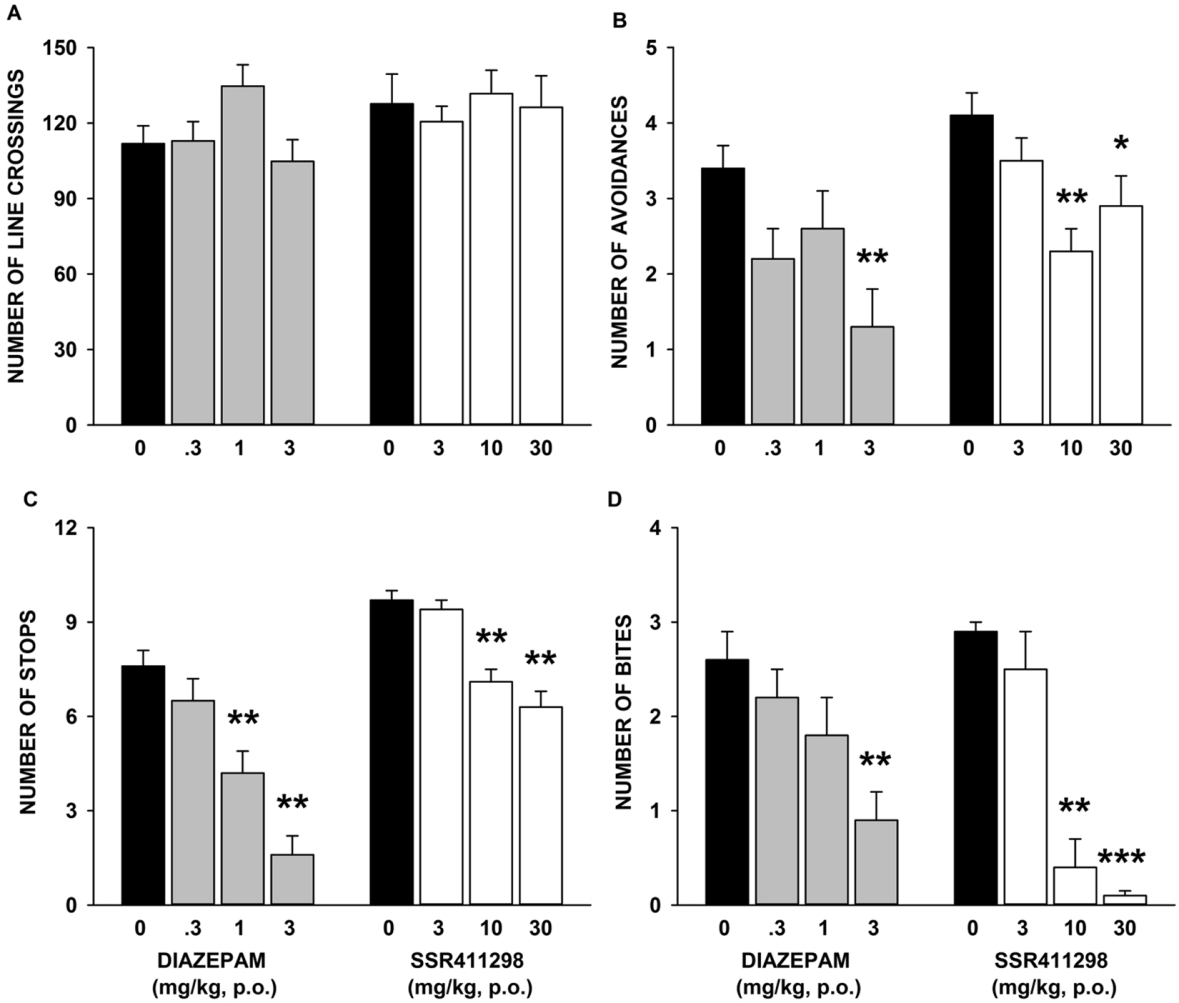
Effects of SSR411298 and diazepam in the mouse defense test battery on (**A**), locomotor activity prior to the exposure to the threat; (**B**) flight response in response to the approaching rat; (**C**) risk assessment when the rat was chasing the mouse, and (**D**), defensive attack reactions upon forced contact with the rat. Data represent mean+SEM, *P < 0.05, **P < 0.01 and ***P < 0.001. N = 7–11 mice per group.

Before exposure to the threat stimulus, neither SSR411298 (F = 0.203, P = 0.89) nor diazepam (F = 2.836, P = 0.052) affected significantly line crossings. In the avoidance test, SSR411298, at 10 and 30 mg/kg, and diazepam, at 3 mg/kg, significantly decreased avoidance frequency (SSR411298: KW = 11.53, P = 0.0092; diazepam: KW = 9.13, P = 0.0276). When mice were chased by the rat, SSR411298 reduced significantly the number of stops (KW = 21.15, P < 0.0001) at 10 and 30 mg/kg. Diazepam similarly decreased stops at 1 and 3 mg/kg (KW = 22.37, P < 0.0001). Upon forced contact with the rat, SSR411298 significantly attenuated bites (KW = 22.45, P < 0.0001) at 10 and 30 mg/kg, and diazepam at 3 mg/kg (KW = 11.69, P = 0.0085). The data are shown in Fig. 9.

#### Social-defeat stress-induced anxiety in the elevated plus-maze test in mice

**Figure 10:**
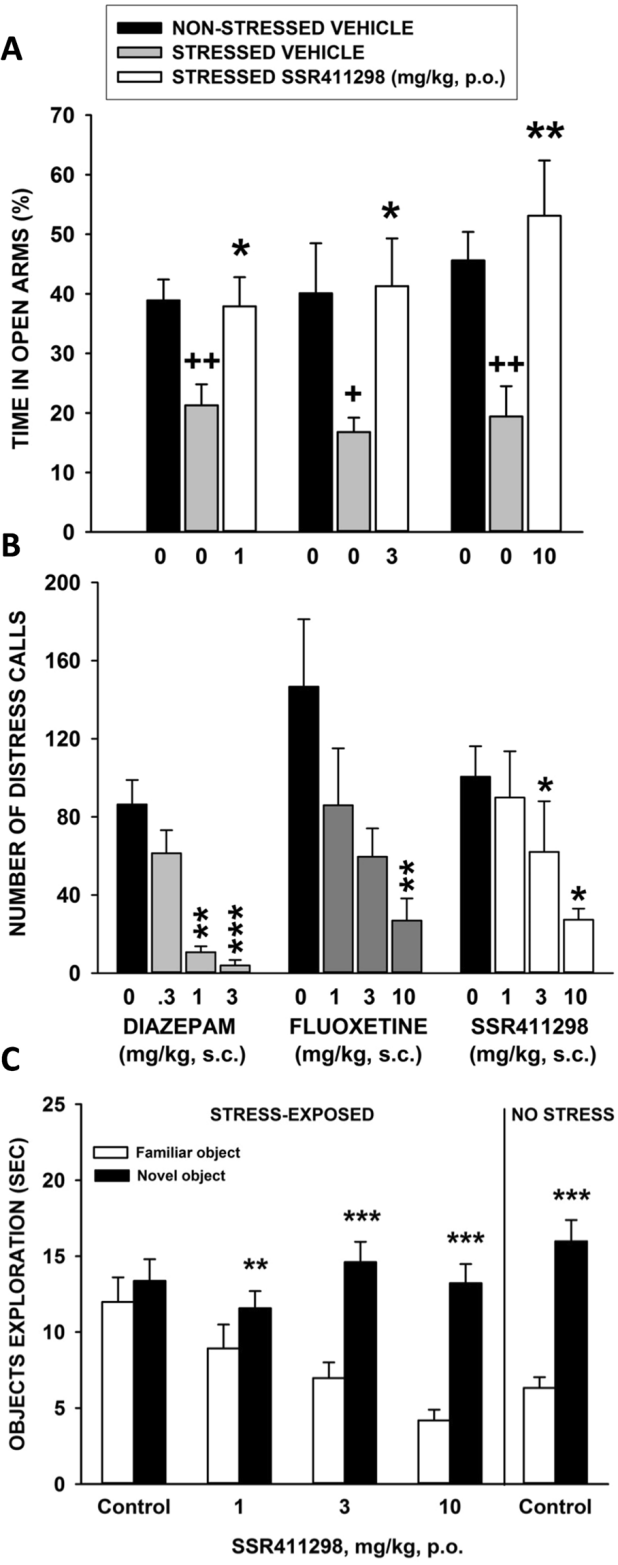
Effects of SSR411298 in several models of anxiety involving prior stress exposure, including (**A**) social defeat induced anxiogenic-like behavior in mice in the elevated plus-maze test. *P < 0.05, **P < 0.01 (vs stressed vehicle) and +P<0.05, ++P < 0.01 (vs non-stressed vehicle). N = 8–13 mice per group; (**B**) ultrasonic distress vocalizations emitted during three minutes by 7-day-old rat pups separated from their mother and littermates. *P < 0.05, **P < 0.01 and ***P < 0.001. N = 7–10 rat pups/group; (**C**) short-term memory impairment induced by inescapable stress in an object recognition task mice. **P < 0.01, ***P < 0.001, new vs familiar object. N = 10 mice per group. Data represent mean+SEM.

In the 3 experiments, statistical analysis demonstrated that social defeat affected significantly the behaviors of animals in the elevated plus-maze, as shown by the decrease in % of time spent in the open arms in stressed animals compared to non-stressed control mice (Wilcoxon tests: P < 0.05). This effect of stress was significantly prevented by SSR411298 at all doses tested (Fig. 10A). The effects of stress and SSR411298 have not been contaminated by alterations in locomotor activity as shown by the lack of significant effect on the number of closed arm entries across experiments (Supplementary Material Table S2). The number of total arm entries, an index which was demonstrated to relate to both anxiety and locomotor activity^47^ was significantly decreased by stress in the experiment investigating 10 mg/kg of SSR411298 (Wilcoxon tests: P < 0.01). The drug normalized this deficit as shown by the lack of significant effect on total arm entries between non-stressed animals and those treated with SSR411298 (Supplementary Material Table S2).

#### Separation-induced ultrasonic distress vocalizations in rat pups

SSR411298 significantly decreased distress calls [χ2 (3) = 9.46, P = 0.0237] at 3 and 10 mg/kg. Diazepam produced a decrease from 1 mg/kg [χ2 (3) = 23.10, P < 0.0001] as did fluoxetine at 10 mg/kg [χ2 (3) = 10.99, P = 0.0118] (Fig. 10B).

#### Stress-induced impairment in episodic memory in the object recognition test in mice

A two-way ANOVA performed on the ranked data revealed a nonsignificant “Treatment” effect [F(4,44) = 1.731, P = 0.1602], a significant “Object” effect [F(1,44) = 116.421, P < 0.0001], and a significant “Treatment” × “Object” interaction [F(4,44) = 7.938, P < 0.0001]. Post-hoc analyses showed no significant difference in exploration time between familiar and novel objects in vehicle-treated stressed animals, while in all other groups (whether stressed or not) that received 1, 3 or 10 mg/kg of SSR411298, animals spent significantly more time exploring the novel object (Fig. 10C).

### Effects in models of depression

#### Chronic mild stress (CMS) test in BALB/c mice

**Figure 11:**
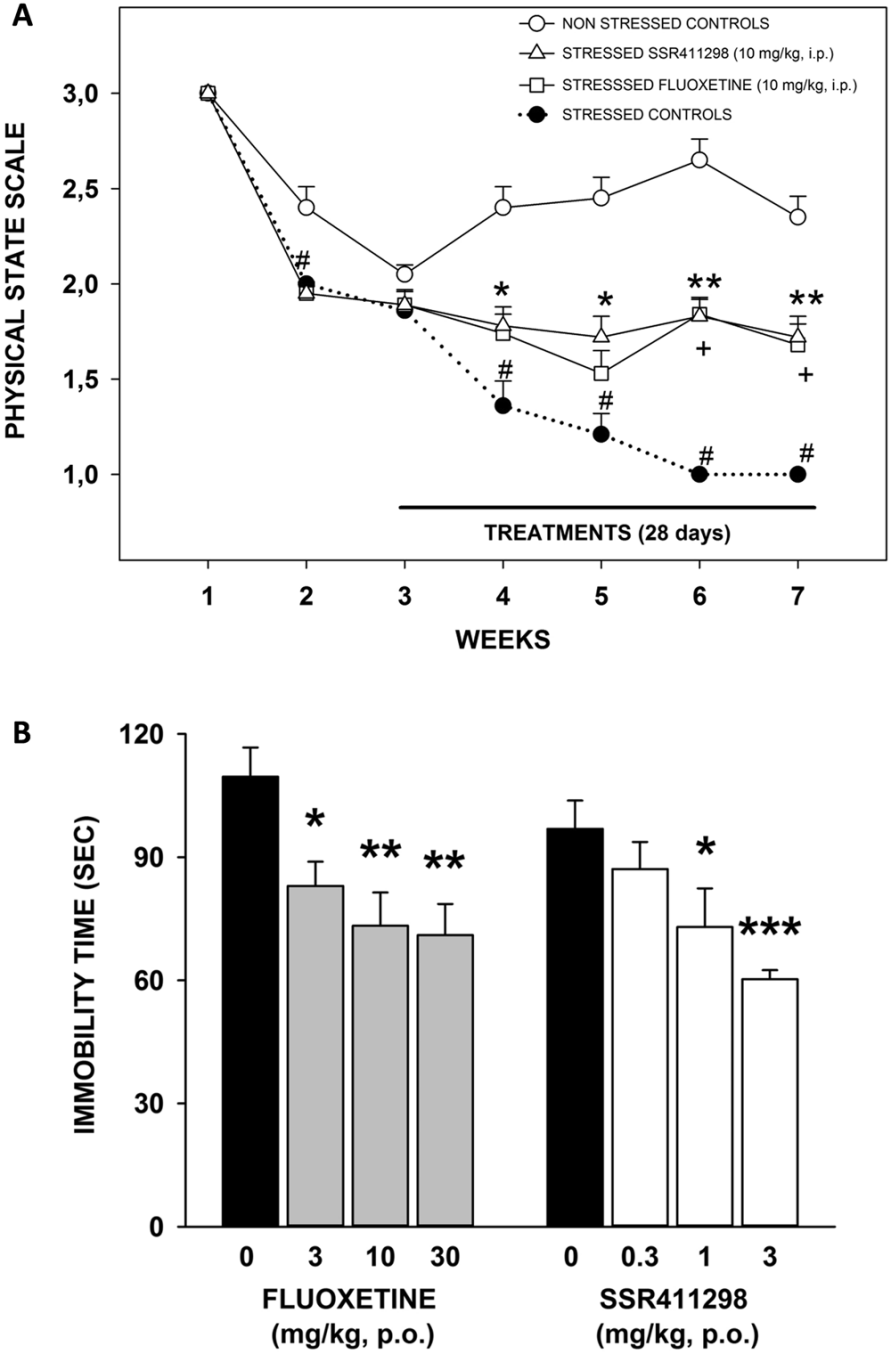
Effect of SSR411298 and fluoxetine in two models of depression: (**A**) the chronic mild stress procedure in mice. Data are expressed as mean physical state score ± SEM. ^#^P < 0.01 (Wilcoxon test versus non stressed control group), *P < 0.05, **P < 0.001 (Kruskal Wallis one sided upper multiple comparison tests with Bonferroni-Holm correction versus stressed control group). N = 14 to 20 mice per group; (**B**) the forced-swimming test in rats. SSR411298 was administered p.o. twice a day during 2 days, 60 minutes before pretest, immediately after, and 60 minutes before the second session of testing. Fluoxetine was administered p.o. twice (immediately after the first session, and 60 minutes before session 2). Data represent mean+SEM. *P < 0.05, **P < 0.01 and ***P < 0.001. N = 6–7 rats per group.

Results showed a significant degradation in the physical state of the coat produced by stress at week 2 and from week 4 to week 7 (Wilcoxon: at least P < 0.05 for each week (Table 3 and Fig. 11A). This effect was improved by SSR411298 at 10 mg/kg from week 4 and by 10 mg/kg of fluoxetine at week 4 and from week 6. These effects lasted until the CMS was over (Fig. 11A). Chronically stressed mice displayed significantly more avoidances when exposed to the rat in the MDTB (Z = −3.11, P = 0.0019) compared to non stressed control animals, an effect which was significantly attenuated by SSR411298 and fluoxetine (χ2 = 11.98, P = 0.0025) (Table 3). In this test, stressed mice failed to display a normal coping behavior showing significantly more bites upon forced contact with the rat than non stressed control animals (Z = −2.86, P = 0.0042) (Table 3). This behavior was fully restored by SSR411298 and fluoxetine, as levels of biting reached those of non stressed control mice (χ2 = 9.33, P = 0.0094) (Table 3). When measured before the exposure to the rat, locomotor activity of stressed animals was increased compared to non stressed controls (t = −4.79, P = 0.0005) (Table 3). This alteration in locomotor activity was not present in stressed mice treated with SSR411298 and fluoxetine; and both groups significantly differed from stressed control group (F = 6.65, P = 0.0044) (Table 3).

**Table 3:** Effects of repeated administration of SSR411298 and the antidepressant fluoxetine for 33 days on chronic mild stress-induced anxiogenic-like behaviors in the mouse defense test battery.

	Non stressed control	Stressed control	SSR411298 (10 mg/kg, i.p.)	Fluoxetine (10 mg/kg, i.p.)
Line crossings	40,9 ± 5,9	82,9 ± 6,5^†^	50,9 ± 5,3**	52,3 ± 9,9**
Avoidances	1.3 ± 0.4	3,5 ± 0,4^†^	1.4 ± 0,6**	0,9 ± 0,4**
Bitings	1,5 ± 0,3	2,8 ± 0,2^†^	1,4 ± 0,5*	1,5 ± 0,3*

Data represent mean ± SEM; **P < 0.01 (vs. stressed control, Kruskal-Wallis or Dunnett’s t-test); ^†^P < 0.01 (vs^.^ non stressed mice, Wilcoxon test). N = 14 to 20.

#### Forced-swimming test in rats

ANOVA indicated significant differences between SSR411298-treated groups and its vehicle-treated control group [F(3,23) = 6.19, P = 0.0031] and between fluoxetine-treated groups and its vehicle-treated control group [F(3,24) = 6.0, P = 0.0034]. Dunnett's post-hoc test indicated that SSR411298 significantly decreased immobility time at 1 and 3 mg/kg, while fluoxetine decreased the behavior at 3, 10 and 30 mg/kg (Fig. 11B).

## Discussion

Enhancing eCB signaling has been proposed to represent a potential strategy for the treatment of several conditions, including psychiatric and neurological disorders, gastrointestinal inflammation, cancer and pain^53^. Here we describe the detailed characterization of SSR411298, a highly selective, brain penetrant and orally-active reversible inhibitor of FAAH.

### Characterization of the mechanism of action of SSR411298

The present results show that SSR411298 has outstanding selectivity for FAAH. Animals treated acutely with SSR411298 show clear inhibition of FAAH in the absence of effect on MAGL^27^ that correlated with ~5-, 13- and 26-fold elevations in endogenous AEA, PEA and OEA, respectively, but not 2-AG levels in the hippocampus. It would be interesting to verify in future studies whether the elevation in these eCBs following SSR411298 is maintained upon chronic treatment. The finding that PEA and OEA concentrations increased to a much greater extent than AEA has been observed in previous studies using FAAH inhibitors^54^. The effects of SSR411298 on AEA levels were confirmed in a behavioral experiment showing that the drug potentiated turning induced by intra-striatal infusion of AEA. It is important to note that the inhibition of FAAH activity following SSR411298 was maintained for a substantial period of time (≥16 hours), but was reversible and slowly returned to baseline by 24 h post-treatment. In general, irreversible enzyme inhibitors, due to their inherent reactivity, typically display limited target selectivity with the potential risk for producing unwanted off-target effects which limits their therapeutic use^15,55^. Only a few completely reversible inhibitors of FAAH have been disclosed, like the α-ketoheterocycle OL-135^56^, the enol carbamate ST4070^57^, and the azetidine analogue V158866^58^. The first in-man study with V158866 showed that the drug had a good tolerability profile. In line with these findings are the results from twelve single or multiple oral dose tolerability, PK and efficacy studies in man showing that SSR411298 is well tolerated (personal communication).

To verify whether elevation in FAAH produced behavioral effects which are reminiscent of CB1 receptor activation, we investigated SSR411298 in a series of experiments aimed at revealing potential cannabinoid behavior. Our findings from the tetrad test show that SSR411298 did not produce the whole spectrum of pharmacological effects classically observed with CB1 receptor agonists. Doses that maximally blocked FAAH activity (≥1 mg/kg, p.o.) produced no catalepsy, ataxia or hypomotility, three typical signs of CB1 receptor activation^59^. However, the drug produced anti-nociceptive effects in the PBQ test, which measures visceral pain sensation, and produced moderate hypothermia. In the drug discrimination experiment where rats were trained to discriminate the CB1 receptor agonist, WIN 55,212-2, from vehicle, SSR411298 did not substitute for WIN 55,212-2 up to a dose as high as 500 mg/kg, p.o., while the cannabinoid Δ^9^-THC substituted for the synthetic CB1 receptor agonist. These findings agree with those of previous studies using different FAAH inhibitors showing that these compounds do not produce CB1-related discriminative-stimulus effects^60,61^. Moreover, in a mouse model for cannabinoid dependence^28^, the CB1 receptor antagonist rimonabant did not induce a precipitated withdrawal syndrome in animals treated with SSR411298 twice a day for 4 days, while a similar treatment regimen with Δ^9^-THC promptly precipitated a profound withdrawal syndrome, including an increase in ptosis and paw tremors, along with a decrease in normal behavior such as licking, scratching and grooming. Taken together, these findings confirm those of previous studies showing that FAAH inhibition does not produce all the behavioral effects of direct-acting cannabinoid receptor agonists, which is a clear advantage as non-preferred effects (abuse liability) are absent. A mechanistic explanation of why FAAH inhibitors only exert certain effects of CB1 receptor agonists remains elusive. However, the finding that FAAH has a more restricted distribution in the brain - for example, the enzyme is much less present in brain reward circuitry than CB1 receptors^62,63^ - may account at least in part for this difference.

### Characterization of SSR411298 in aversive and non-aversive learning tasks

Substantial evidence from both animal research and human studies indicates that cannabinoids affect learning and memory processes^64^. However, the effects induced by pharmacological modulation of eCB neurotransmission on memory are often contradictory. There have been reports of both deficits in memory and enhancing effects after the administration of direct cannabinoid receptor agonists or inhibitors of eCB catabolism and transport^65^. Among the different factors involved is the level of emotional arousal induced by the experimental conditions. Administering eCB-enhancing drugs in proximity to extrinsic stress exposure generally facilitates memory or normalizes stress-induced memory impairment. In contrast, in non- or low aversive learning tasks, pharmacological activation of the eCB system tends to impair memory acquisition and consolidation. In this study, we show that oral administration of SSR411298 had no effect on acquisition and consolidation in non-aversive tests addressing different aspects of hippocampal-dependent memory, such as working or episodic memory. Studies that investigated the effects of FAAH inhibitors (mainly URB597) in learning and memory models with no extrinsic stress revealed contrasting results, with some studies showing impairment^66–68^, while other demonstrated improvement of memory function^33,69,70^. Possible confounding factors that have been suggested to explain these contrasting results include the use of different environmental and experimental conditions, the time of drug administration (pre- versus post-training, and pre- versus post-retrieval administration) and drug dose levels^71^. Here, SSR411298 was tested in memory tasks over a wide dose-range and administered prior to acquisition or retrieval phase, treatment schedules which have been used to show memory deficits of FAAH inhibitors in previous studies. It can therefore be excluded that these two latter factors may explain the lack of memory-impairing effect of SSR411298. It is also worth noting that our learning tasks are insensitive to enhancement effects. In previous studies, we did not observe memory enhancement with drugs known to produce cognitive-enhancing effects in other procedures^34,72^. Additional experiments are necessary to determine whether SSR411298 may have memory-enhancing properties *per se*.

As indicated above, when cannabinoid enhancing drugs are given in proximity to an environmental stressor (shortly before or after exposure to stress), they can attenuate the action of stress on learning and memory^65^. Here we used the object recognition task in mice previously exposed to a stressor (i.e. a pair of rats) to determine if SSR411298 is able to normalize the deficit of memory performance induced by stress. The results show that SSR411298 administered prior to acquisition attenuated completely the deleterious effects of stress on visual episodic memory performance. It should be emphasised that the cognitive enhancer donepezil, which was tested previously in this procedure, did not improve performance in stressed mice^73^, indicating that the effects of SSR411298 cannot be explained by potential cognitive-enhancing effects of the drug, but rather by a specific action on emotional memory. Previous studies using pharmacological inhibition or genetic deletion of FAAH have demonstrated enhanced memory performance in rodents trained with procedures involving aversively motivated behavior or using extrinsic stress manipulations^70,74–77^. For example, FAAH^−/−^ mice and mice treated with OL-135 exhibited an increase in the acquisition and extinction rates in the Morris water maze test^77^. Other studies showed that systemic administration of AM3506 as well as local application of URB597 into the medial prefrontal cortex prior to extinction training improved extinction retrieval in rodent models of impaired fear extinction^78,79^. The present findings with SSR411298 showing improved memory performance after stress exposure are in line with these studies and support further the idea that FAAH inhibitors may represent a valid therapeutic option to treat traumatic fear memories.

The mechanisms underlying cannabinoid modulation of emotional memory *via* inhibition of FAAH remain to be fully elucidated. It was proposed that these effects are mediated by a CB1-dependent signaling mechanism in the basolateral amygdala (BLA): as a result of FAAH inhibition, the elevation of AEA in the BLA increases CB1 receptor signaling to inhibit GABAergic transmission. This in turn removes an inhibitory brake on BLA output neurons necessary for the encoding of emotional memories^80^. Additionally, it was suggested that the reduction in GABAergic activity in the BLA as a consequence of CB1 receptor activation after stress exposure can decrease the activation of the hypothalamic-pituitary-adrenal stress axis (and the release of corticosterone) and modulate the effects of aversive events on emotional memory^65^. More recently, it was demonstrated that the modulation of aversive memory by URB597 is dependent not only upon CB1 receptor activation but additionally involves PPAR-α and TRPV1 receptor activation^71^.

### Characterization of SSR411298 in models of anxiety and depressive disorders

Much attention has focused in recent years on the eCB system as a potential target for novel anxiolytics^12,81^. AEA and CB1 receptors are densely expressed in brain regions mediating anxiety^82^. Moreover, there is compelling evidence that changes in CB1 receptor signalling and other eCBs are involved in anxiety conditions such as PTSD^78,83,84^. The effects of directly-acting CB1 receptor ligands on anxiety-like behaviors have been widely assessed across a range of experimental models in animals, with mixed results, which have been tentatively explained by the ubiquitous presence of CB1 receptors in various anxiety-mediating brain circuits and areas, some of which may have opposing roles in anxiety^35^. Since eCBs are mainly released ‘on demand’ as a function of physiological requirements, it was suggested that the pharmacological inhibition of their degradation could increase functionally relevant recruitment of eCBs and, as a result, more selectively modulate anxiety behaviors than CB1 receptor agonists^35^. Here we show that acute administration of SSR411298 produced anxiolytic-like effects in some, but not all models used. The drug was completely inactive over a wide dose-range in the light/dark test in mice and the punished drinking test in rats, two classical models of anxiety. In the social interaction task, SSR411298 reduced anxiety-like behaviors as demonstrated by an increased social interaction in dyads of unknown gerbils. However, the magnitude of the effect was less than that of diazepam, a prototypical benzodiazepine anxiolytic. Whether this finding suggests a less efficacious anxiolytic-like potential of SSR411298 compared with benzodiazepines, or indicates that the FAAH inhibitor may have a different spectrum of therapeutic activity in anxiety conditions than benzodiazepines, remains to be verified. Findings from the MDTB may, however, be relevant to this issue. Previous experiments using this test battery have suggested that this procedure provides a model able of responding to, and differentiating anxiolytics of various classes through specific profiles of effect on different behaviors^38^. In the present study, SSR411298 only weakly modified flight and risk assessment, behaviors that have been demonstrated to be more sensitive to benzodiazepines or selective 5-HT reuptake inhibitors, i.e., molecules generally used against generalized anxiety disorder (GAD) and panic disorder (PD). However, it produced marked effects on defensive aggression, a terminal defense reaction that may relate to certain aspects of stress disorders after traumatic events^85^, suggesting that SSR411298 may be useful in these conditions rather than in PD or GAD. In line with this hypothesis are the findings from the social defeat test, where SSR411298 normalized the heightened anxiety-like behaviors in the EPM following (stressful) exposure to an aggressive resident. Further in line with this idea, we found that SSR411298 attenuated distress vocalizations of rat pups separated from their mother and normalized stress-induced memory impairment in the object recognition task in mice. Together, these data in anxiety models suggest that SSR411298 may be preferentially active under conditions of high stress, a finding that adds further strength to an already substantive body of evidence that genetic deletion or pharmacological blockade of FAAH produced anxiolytic-like effects more reliably under conditions of high environmental aversiveness (for a recent review, see^12^).

The mechanisms underlying the specificity by which FAAH inhibitors only exert anxiolytic effects under highly aversive conditions remain to be fully elucidated. A current hypothesis indicates that FAAH inhibition, and resultant increase of AEA in brain regions involved in the regulation of anxiety, results in the restoration of dysfunctional homeostasis of AEA signaling as a consequence of stress exposure^12^. However, additional mechanisms underlying the effects of FAAH inhibitors in anxiety tests have been proposed involving CB1-mediated modulation of glutamate in the central amygdala^86^ and of serotonin in the hippocampus^87^.

Regulation of eCB signaling has been implicated in the pathophysiology of MDD^88^. For example, in animal studies, prolonged stress was reported to compromise eCB signaling in stress-responsive neural circuits leading to poor habituation to stress, which in turn led to the development of maladaptive behaviors, such as anhedonia, which are reminiscent of symptoms of depression^89^. In humans, patients with MDD showed decreased serum levels of AEA as compared to healthy controls^90^. These and other findings (reviewed by Ogawa and Kunigi^13^) have led to the idea that potentiation of eCB signaling may represent a valid approach for the development of novel antidepressants. To investigate potential antidepressant-like effects of SSR411298, we used the CMS and the forced swimming tests, two classical chronic and acute models of depression, respectively. In the forced swimming test, SSR411298 displayed antidepressant-like activity comparable to those of the reference antidepressant, fluoxetine. The antidepressant-like potential of the FAAH inhibitor was confirmed in the CMS test following repeated administration of the drug for 33 days. SSR411298 improved the degradation of the physical state of the coat of stressed animals, suggesting that the drug normalized grooming, an activity impaired by repeated stress. In addition, stress coping was impaired by CMS. It caused the appearance of an anxious profile in the MDTB. These behavioral changes were absent in mice that received SSR411298, indicating that the drug restored a normal anxiety level. Further, in the MDTB chronically stressed animals failed to show normal locomotor activity under an unfamiliar environment, a behavior which was normalized by SSR411298. Taken together, these findings indicate that SSR411298 exerts robust antidepressant-like activity, restoring notably the development of maladaptive behaviors to chronic stress. These results are line with previous studies showing that FAAH inhibitors produce antidepressant-like effects in a variety of animal models and further build upon the idea that FAAH inhibition may represent an alternative avenue for the development of antidepressants (reviewed by Ogawa and Kunugi^13^).

While it cannot be excluded that at least part of the mechanisms underlying the antidepressant-like effects of FAAH inhibitors overlap with those responsible for their anxiolytic-like effects^13,87^, several authors have highlighted the importance of AEA signaling at CB1 receptors in the prefrontal cortex and the hippocampus in these effects. It was notably proposed that the downstream mechanisms of FAAH inhibitors converge with those of 5-HT-acting antidepressants, including modifications of hippocampal 5-HT receptor function, and enhancement in BDNF production in the hippocampus and prefrontal cortex^87,91^.

## Conclusion

In conclusion, the present experiments demonstrated that the selective, orally-active and reversible FAAH inhibitor, SSR411298, shows efficacy in models of emotional disorders that are precipitated by stress. The drug is safe and does not mimic the interoceptive state or produce the behavioral side-effects evoked by direct-acting cannabinoids, including impairment in motor activity, physical dependence, and deficits in learning and memory. This drug profile positions SSR411298 as a promising candidate for the treatment of conditions associated with the development of maladaptive behaviors, including those seen for example in PTSD and MDD.

## Electronic supplementary material

Supplementary Dataset 1
