# Supplementary material for: The selective reversible FAAH inhibitor, SSR411298, restores the development of maladaptive behaviors to acute and chronic stress in rodents
Source: Sci Rep. 2018 Feb 5;8:2416. doi: 10.1038/s41598-018-20895-z (PMC5799259; doi:10.1038/s41598-018-20895-z)
Supplement: Supplementary file 1 — Supplementary Dataset 1 [file 41598_2018_20895_MOESM1_ESM.docx]

**SUPPLEMENTARY MATERIAL**

**The selective reversible FAAH inhibitor, SSR411298, restores the development of maladaptive behaviors to acute and chronic stress in rodents**

Guy Griebel, Jeanne Stemmelin, Mati Lopez-Grancha, Valérie Fauchey, Franck Slowinski, Philippe Pichat, Gihad Dargazanli, Ahmed Abouabdellah, Caroline Cohen & Olivier E. Bergis

**Table S1**: The GPCR, ion channel and enzyme panel

| GPCRs and channels | 5-HT_1B_, 5-HT_1D_, 5-HT_2A_, 5-HT_2C_, 5-HT_3_, 5-HT_4E_, 5-HT_6_, 5-HT_7_, 5-HT transporter, A_1_, A_2A_, A_3_*,*α_1_ (non-selective), α_2A_, anandamide transporter, AR, AT_1_, AT_2_, BZD (central), BZD (peripheral), β_1_, β_2_, BB (non-selective), B_1_, B_2_, Ca^2+^ channel (N), Ca^2+^ channel (L, dihydropyridine site), Ca^2+^ channel (L, verapamil site) (phenylalkylamine), CB_1_, CB_2_, CCK_1_ (CCK_A_), CCK_2_ (CCK_B_), Cl^-^ channel, CCR_1_, CCR_2_, CXCR_2_ (IL-8B), D_1_, D_2_, DA transporter, ERα, ETA, GABA_A_, GABA_B_(1b), GABA transporter, GAL_1_, GAL_2_, glycine (strychnine-sensitive), GR, kainate, K^+^v channel, KATP channel, κ (KOP), H_1_ (peripheral), H_2_, H_3_, H_4_, IL-8, M_1_, M_2_, MCH_1_, MC_3_, MC_4_, μ, N (neuronal), N neuronal α-BGTX-insensitive (α4β2), Na^+^ channel (site 2), NE transporter, NMDA, NK_1_, NK_2_, NK_3_, Y_1_,Y_2_, NTS_1_ (NT_1_), NMU_2_, δ_2_ (DOP), NOP (ORL_1_), P2X, P2Y, PCP, PR, σ (non-selective), sst (non-selective), TNF-α, TR (TH), V_1a_ *,*V_1b_, V_2_, VPAC_1_ (VIP_1_), VR_1_ |
| --- | --- |
| Enzymes | 12-lipoxygenase, ACE, arachidonate 5-lipoxygenase, CaMK2α, cathepsin D, cathepsin L, constitutive NOS (endothelial), cholinesterase, COX1, COX2, cPLA2, elastase, Factor VII, IX, X, IRK (InsR), MAO-B, MMP-1, PDE4D, phosphatase 1B (PTP1B), plasmin, PLC, thrombin, TPA, trypsin, tryptase |

**Table S2**: Effects of SSR411298 on measures related predominantly to activity in the elevated plus-maze following social defeat in mice.

|  | Closed arm entries | Total entries |
| --- | --- | --- |
| Non-stressed vehicle | 9,8±1,39 | 17,4±2,19 |
| Stressed vehicle | 10,5±1,41 | 16,1±2,19 |
| Stressed SSR411298 (1 mg/kg, p.o.) | 10,2±1,5 | 18,8±2,5 |
|  |  |  |
| Non-stressed vehicle | 5,0±0,77 | 9,6±1,33 |
| Stressed vehicle | 8,0±1,49 | 12,0±2,0 |
| Stressed SSR411298 (3 mg/kg, p.o.) | 5,4±1,16 | 10,7±1,86 |
|  |  |  |
| Non-stressed vehicle | 7,1±0,74 | 13,5±0,78 |
| Stressed vehicle | 5,1±1,03 | 8,1±1,31++ |
| Stressed SSR411298 (10 mg/kg, p.o.) | 5,1±1,27 | 11,3±1,9 |

++P<0.01 (vs non-stressed vehicle). N=8-13 mice per group.

**Figure S1**: Schematic representation of the drug discrimination training and generalization procedures.


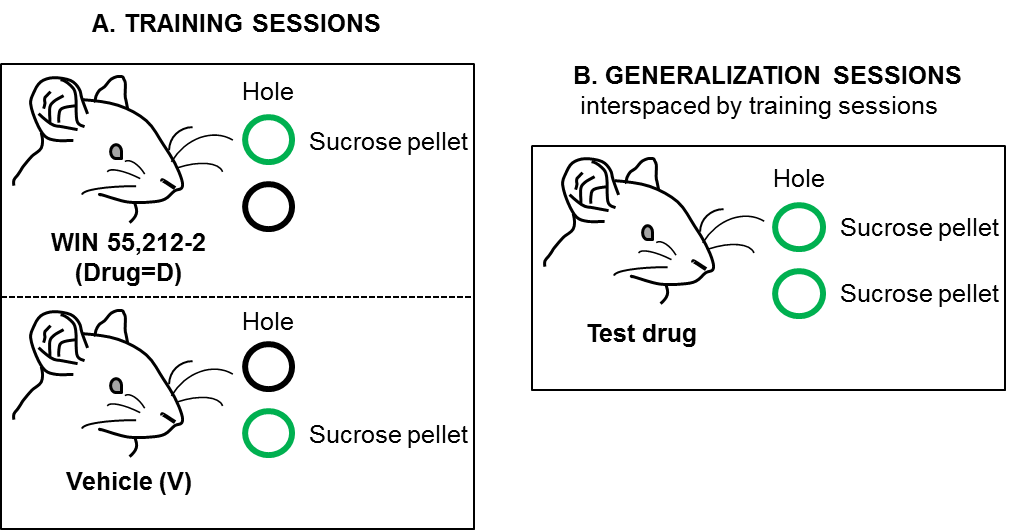


A) During training sessions, half the animals were reinforced with sucrose pellets for responding on the right hole after dosing with WIN 55,212-2 at 0.25 mg/kg and on the left hole after dosing with vehicle. For the second half, these conditions were reversed. The training drug (D) and its vehicle (V) were given in a pseudorandom order. The following pattern V, D, D, V, V, D, V, V, V, D, D was repeated until the criteria were reached by each animal. The criteria were the following: <10 incorrect nose poke prior first sucrose pellet, ≥ 25 pellets per sessions, ≥ 85% responses on correct hole during 5 consecutive or 6 out of 7 sessions.

B) When the criteria were reached for an animal, generalization sessions were conducted with SSR411298 at 10, 30, and 100 mg/kg, its vehicle, Δ^9^-THC at 2 mg/kg, its vehicle, each being administered on a single occasion according to a latin-square design, and completed by a last session with SSR411298 at 500 mg/kg. Responding on either hole was reinforced with sucrose pellet. Generalization sessions were interspaced by at least two training sessions, for which the animal was required to satisfy criteria for one training sessions with WIN 55,212-2 and one with its vehicle.
